# Implications of Ho^3+^-ions on physical, structural, optical, and spectroscopic features of Na_2_O-PbO-borotellurite glass system for feasible applications in optical and laser technology

**DOI:** 10.1038/s41598-025-13648-2

**Published:** 2025-08-01

**Authors:** Shiva Kumar B N, Devaraja C, R. S. Gedam

**Affiliations:** 1 Department of Physics, Manipal Institute of Technology Bengaluru, Manipal Academy of Higher Education, Manipal, 576104 Karnataka India; 2 Department of Physics, Visvesvaraya National Institute of Technology, Nagpur, 440010 Maharashtra India

**Keywords:** Borotellurite glass, Holmium, Energy band gap, Refractive index, Metallization criterion, And photoluminescence, Condensed-matter physics, Materials for optics, Materials science

## Abstract

A novel series of Holmium-ions doped Na_2_O-PbO-borotellurite glasses with composition yHo_2_O_3_+(65-y)B_2_O_3_ + 15TeO_2_ + 10Pb_3_O_4_ + 10Na_2_O, where $\:\text{y}=$ 0.0 to 0.5 mol% with 0.1 increment, have been prepared by the melt-quenching approach. The non-crystalline state of the synthesized glasses was validated through the XRD technique. The structural modifications and presence of borate networks (BO_3_ and BO_4_), tellurite networks (TeO_3_ and TeO_4_), and Pb-O links were confirmed through ATR-FTIR and Raman spectroscopic methods. The morphology and analysis of elements were performed by employing the SEM-EDX technique. The optical characterizations were studied through UV-visible spectroscopy. The transitions ^5^I_8_ → ^5^G_5_, ^5^G_6_, ^5^F_1_+^5^G_6_, ^5^F_2_, ^5^F_3_, ^5^S_2_+^5^F_4_ and ^5^F_5_ were noted. Emission spectra of the Ho^3+^-ions incorporated glasses were obtained using a spectrofluorometer at an excitation wavelength of 451 nm, and they exhibit an intense peak at 678 nm in the region of red. The CCT values found $\:<\:$4000 K imply a warm CCT. The merits of physical and optical parameters viz., molar volume, density, ion concentration, field strength, optical band gaps, refractive index, Urbach energy, steepness parameter, molar polarizability, metallization criterion, electronic oxide polarizability, optical basicity etc., were evaluated using appropriate relations and they found to be in comply with the structural modifications and optical features. The density and refractive index are found between 4.067 gcm^−3^ to 4.193 gcm^−3^ and 2.345 to 2.367, respectively. The energy band gaps and Urbach energy range from 3.115 eV to 3.201 eV and 0.247 eV to 0.269 eV, respectively. Optical basicity and electronic oxide polarizability range from 1.254 to 1.271 and 4.015 Å^3^ to 4.178 Å^3^, respectively. Metallization criterion values range from 0.395 to 0.401. Based on structural, optical, luminescence properties, and the physical and optical parameter merits, the synthesized glasses can be suitable for optical devices, including lasers, red phosphors, and optical amplifiers.

## Introduction

 Glass, a versatile and transformative material, has undergone significant advancements over the past decades, leading to its widespread applications in various industries such as telecommunications, energy, healthcare, electronics, and construction. Novelties in composition, fabrication methods, and functionality have resulted in glass with a substantial enhancement in structural^1,2^, optical^2,3^, thermal^2,4^, luminescence^5–8^, and electrical^9–11^ properties. Innovations in optical and laser technology^12–16^, bioactive materials^17,18^, and sustainable production processes are driving the future of glass applications^19,20^. In recent years, tellurium dioxide (TeO_2_) based glasses have gained scientific and technological attention due to their unusual features, including distinctive characteristics, for instance, relatively lower melting temperatures and phonon energy thresholds, excellent thermal and chemical stability, reduced crystallization tendencies, and enhanced refractive indices. Additionally, tellurium oxide-based glasses are regarded as intriguing materials for optical amplifiers owing to the notable third-order nonlinear susceptibility^12,21–28^. Pure TeO_2_ is inadequate to form glass under typical circumstances^29^. However, under certain conditions, the incorporation of various oxides widens the glass-forming range. When TeO_2_ combines with B_2_O_3_, which is a basic glass-forming due to its longer bond length, smaller cation size, and trivalence of boron, boro-tellurite glasses are formed. Currently, borotellurite glasses have gained prominence for their uses and improvements in the quality, transparency, and refractive index. Boro-tellurite glasses are currently considered to be superior materials for advanced photonic and optoelectronic applications^30^, including optical communications and high-performance lasers^2,13–15,31^ because of their low phonon energy (~ 800 cm^−1^) and suitable host to rare earth elements^5,15,16,32–34^.

Heavy metal oxides (HMOs) such as lead oxide (PbO) serve as glass network modifiers in borate and tellurite-based glasses^35–39^. When PbO is added to the glass matrix, it is typically introduced as a modifier. It disturbs the network by disrupting boron-oxygen and tellurium-oxygen connections, resulting in non-bridging oxygens (NBOs), which open the structure and affect the glass’s physical qualities. Its high atomic number and polarizability enhance the refractive index of glass, which is significant in optical applications, including optical fibers and lenses. When coupled with rare-earth dopants, PbO increases glass density and light interaction and enhances glass luminescence via sensitizing rare-earth ions, resulting in increased emission qualities intended for solid-state lasers and photonic applications and also radiation shielding applications^6,40,41^. The inclusion of alkali metal ions (Na, Li, etc.) could improve the rare earth ions’ solubility, modify the glass network by converting BO_3_ to BO_4_ units, and aid in reducing the phonon energy and improve the chemical durability of B_2_O_3_-TeO_2_ glasses^42^.

Rare earth (RE) elements such as Europium, Holmium, Praseodymium, etc., have revolutionized technology due to their unique features and are considered the backbone of modern technology, playing a crucial role in energy, defence, healthcare, and environmental solutions^43,44^. Their properties enable the advancement of cutting-edge applications, driving innovations across various sectors and paving the way for future technological breakthroughs^45–48^. Currently, focusing on RE-ions-inorganic glasses has gained attention to enhance the optical and physical features of host glasses owing to their distinct spectroscopic features caused by optical transitions in the intra-4f shell. Among the RE-elements, holmium is a prevalent rare earth element in which energy levels facilitate population inversions due to its long-lasting intermediate metastable levels. It exhibits red^49^, green^50^, and near-infrared (IR)^51^ emission and is often employed in visible lasers, radar, sensors, and medical diagnostic tests^7,50,52–54^.

Basappa et al.^55^ explored the optical properties of borate glasses doped with holmium and embedded with Ag_2_O and suggested the resulting glasses for applications in photonics and optoelectronics. Vani et al.^26^studied the structural and optical properties of tellurite glasses doped with holmium and reported that the obtained glasses could be potentially useful for nonlinear optical applications. Ansari et al.^49^ investigated the spectroscopic features of borophosphate glasses doped with holmium and concluded for near-IR light applications.

Meanwhile, Zhou M et al. reported^56^ that among the trivalent RE-ions, the Ho^3+^-ion is one of the intriguing ions for spectroscopic investigations because of the various electronic transitions it may show in the visible and IR range. In addition, the borotellurite glasses doped with holmium, with the inclusion of post-transition elements such as lead, tin, etc., are explored to a much lesser extent for possible applications in various fields, including optics, photonics, and laser technology.

Taking into account the aforementioned considerations, the present study focused on novel glass systems with the series yHo_2_O_3_+(65-y)B_2_O_3_ + 15TeO_2_ + 10Pb_3_O_4_ + 10Na_2_O, where y $\:=$ 0.0 to 0.5 mol%, were synthesized. The physical, structural, optical, and photoluminescence properties were explored for feasible applications in optical and laser technology for industry and innovation purposes.

### Synthesis process and characterization techniques

**Fig. 1:**
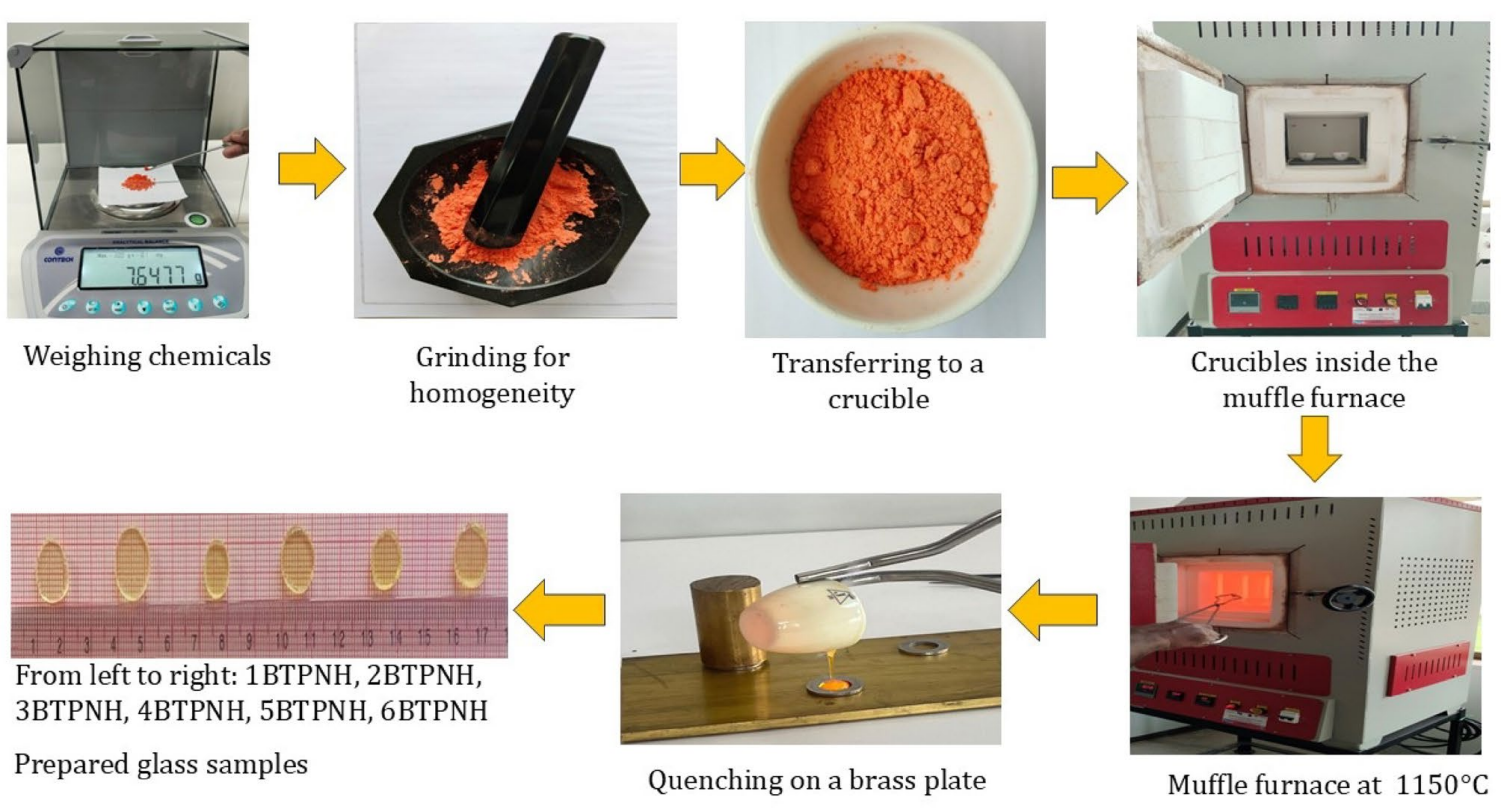
The flow chart shows the preparation of BTPNH glasses.

A new set of holmium oxide-doped Na_2_O$\:-$PbO$\:-$Borotellurite glasses having the composition yHo_2_O_3_+(65-y) B_2_O_3_ + 10TeO_2_ + 10Pb_3_O_4_ + 10Na_2_O, where y = 0.0 mol% − 0.5 mol% of holmium with 0.1 mol% of holmium increment, were prepared by the melt-quenching approach. Hereupon, the synthesized glasses are coded as 1BTPNH, 2BTPNH, 3BTPNH, 4BTPNH, 5BTPNH, and 6BTPNH, which correspond to 0.0, 0.1, 0.2, 0.3, 0.4, and 0.5 mol% of Ho_2_O_3_, respectively. The composition of BTPNH glasses is listed in Table 1. The chemicals H_3_BO_3_, TeO_2_, Pb_3_O_4_, Na_2_CO_3_, and Ho_2_O_3_ of analytical reagent (AR) were accurately weighed according to the required molar mass ratios. The materials were finely ground for 25 min in the agate mortar and pestle to achieve a uniform blend. The resulting powder was formulated according to the intended composition and was placed into a porcelain crucible and then covered with a lid. The crucible was then introduced into a muffle furnace, where the temperature was systematically raised to 1150○C in accordance with the glass composition requirements. This temperature was maintained for 20 min, during which the molten mixture was stirred carefully to achieve homogeneity. Afterward, molten material was cast onto a brass mold and swiftly pressed using a brass weight to avoid crystallization. A schematic flow chart outlining the synthesis steps and formation of final glass samples is illustrated in Fig. 1.

The obtained glasses were taken in bulk and powdered forms, which are suitable for further analysis and characterization. To examine the non-crystalline nature, the Bruker-D8 Focus X-ray diffractometer (XRD) was utilized to record an X-ray diffraction from $\:10^\circ\:\:\text{t}\text{o}\:80^\circ\:.$ To investigate structural changes and ensure the existence of functional groups, Fourier transform infrared (FTIR) spectra were recorded at 500 cm^−1^ to 2000 cm^−1^ using a Bruker-Alpha II spectrometer, and Raman spectra were obtained in the range of 500 cm^−1^ to 2000 cm^−1^ using a Raman microscope with a peak seeker probe pro-Raman system. The morphology and elemental analysis were done through a model-EVO 10 scanning electron microscope and energy-dispersive X-ray spectroscope (SEM-EDX) having a tungsten filament with an acceleration voltage between 0.2 kV and 30 kV. The UV-visible spectra were recorded from 300 nm to 800 nm using a UV-visible spectrometer (Shimadzu, UV-1900I model). Fluorescence emission was explored in the visible region, 550–750 nm, through the JASCO FP-8200 spectrofluorometer. The essential optical and physical parameters are determined and interpreted.

The density ($\:\rho\:$) of BTPNH glasses was evaluated through Archimedes’ principle^23,57^ by measuring the weights of glasses in the air and then in toluene. The formula utilized for the determination of density is

1 $$\:{\uprho\:}=\left(\frac{{\text{W}}_{\text{g}\text{a}}}{{\text{W}}_{\text{g}\text{a}}-{\text{W}}_{\text{g}\text{t}}}\right){{\uprho\:}}_{\text{t}}$$

Where, $\:{\text{W}}_{\text{g}\text{a}}=\:$Weight of glass in air, $\:{\text{W}}_{\text{g}\text{t}}=$ Weight of glass in toluene, and $\:{{\uprho\:}}_{\text{t}}=$ 0.8635 gcm^−3^, the density of toluene.

Molar volume ($\:{\text{V}}_{\text{m}}$) of BTPNH glasses was calculated using the relation^23,57^

2 $$\:{V}_{m}={M}_{av}/\rho\:$$

Where, $\:{\text{M}}_{\text{a}\text{v}}$ is the average molecular weight.

Oxygen packing density $\:\left(OPD\right)$ of BTPNH glasses was computed through the relation^58^

3 $$\:OPD=\frac{\rho\:\times\:a\times\:{10}^{3}}{{M}_{av}}$$

Where, $\:a=$ Number of oxygen atoms per formula unit.

Boron-Boron distance ($\:{d}_{B-B}$) in the BTPNH glasses was computed by the relation^58^

4 $$\:{\text{d}}_{\text{B}-\text{B}}={\left[\frac{{\text{V}}_{\text{m}}^{\text{B}}}{{\text{N}}_{\text{A}}}\right]} ^{1/3}$$

Where, $\:{\text{V}}_{\text{m}}^{\text{B}}=\frac{{\text{V}}_{m}}{1-{\text{X}}_{\text{B}}}$, is the volume of 1 mol of boron atoms, $\:{\text{X}}_{\text{B}}=$ molar fraction of B_2_O_3_ and $\:{\text{N}}_{\text{A}}=\:$6.0223$\:\times\:{10}^{23}$ mol^−1^ Avogadro’s number.

The comprehensive evaluation of the structural, physical, optical, and luminescence properties highlights the potential of glass systems for advanced applications in optics and lasers.

**Table 1:** Composition of BTPNH glasses (mol%).

Sample code	B_2_O_3_	TeO_2_	Pb_3_O_4_	Na_2_O	Ho_2_O_3_
1BTPNH	65	15	10	10	0.0
2BTPNH	64.9	15	10	10	0.1
3BTPNH	64.8	15	10	10	0.2
4BTPNH	64.7	15	10	10	0.3
5BTPNH	64.6	15	10	10	0.4
6BTPNH	64.5	15	10	10	0.5

## Results and discussions

### X-ray diffraction studies

**Fig. 2:**
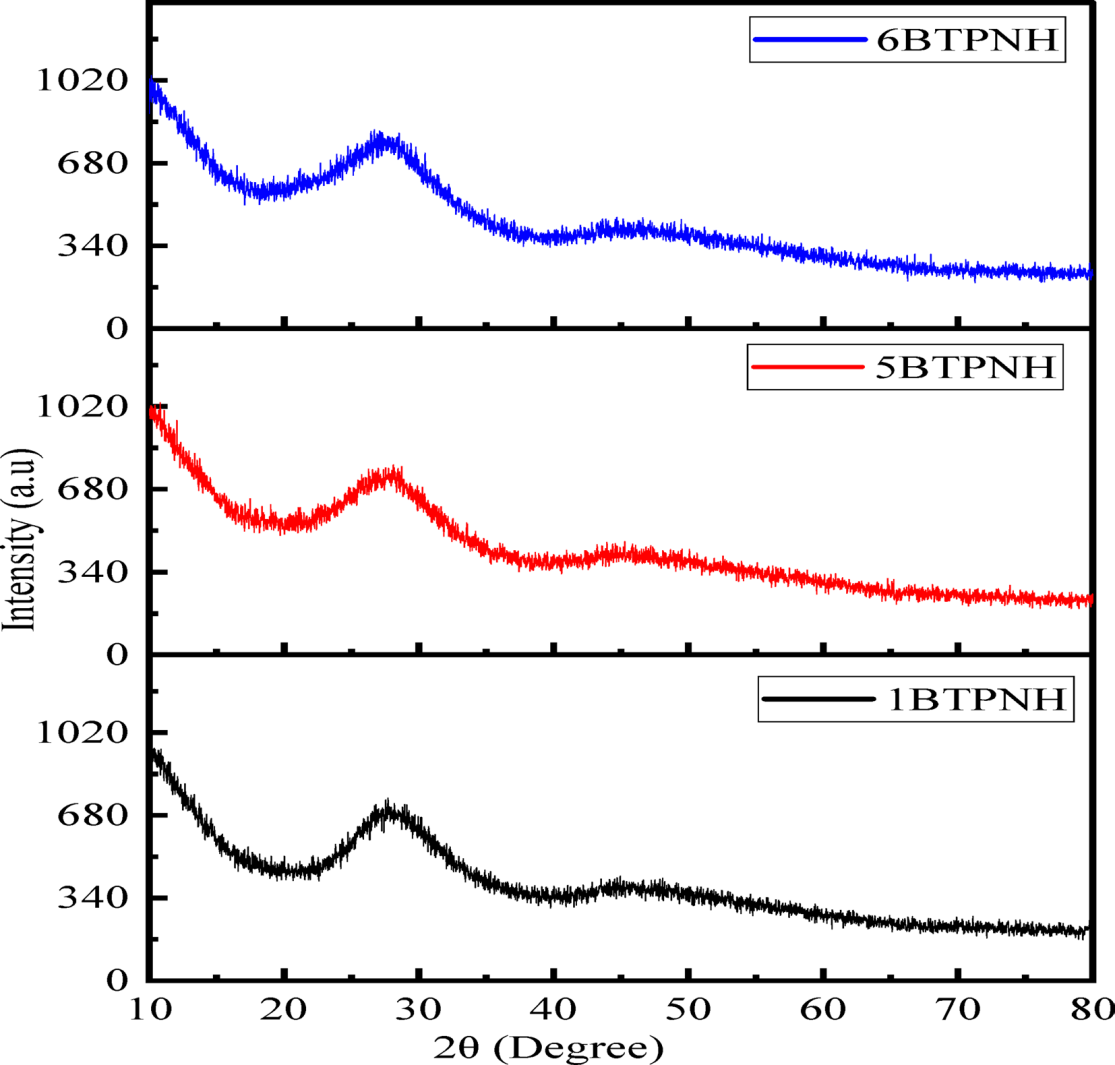
A typical graph of X-ray diffraction profiles of BTPNH glasses.

In general, the amorphous state of glasses was examined through the X-ray diffraction (XRD) technique. So, XRD analysis was performed on the 1BTPNH, 5BTPNH, and 6BTPNH glasses, and the corresponding diffraction pattern is presented in Fig. 2. The diffraction profiles exhibit two broad hump between 20$\:^\circ\:$ and 37$\:^\circ\:$ and 40$\:^\circ\:$ to 57$\:^\circ\:$ due to the simultaneous scattering of atoms and molecules. It has been noticed that the absence of sharp diffraction peaks. This is an indication of a non-crystalline structure with a lack of long-periodic atomic arrangement in BTPNH glasses. This confirmed the glassy nature of synthesized BTPNH glasses^38,59,60^.

### Physical properties

#### Density, molar volume, oxygen packing density, and boron-boron separation

The Eqs. (1), (2), (3), and (4) were used to determine density, molar volume, oxygen packing density, and boron-boron separation. The determined values of density ($\:{\uprho\:}$), molar volume ($\:{M}_{v}$), and boron-boron distance $\:\left({d}_{B-B}\right)$ are listed in Table 2. The density and molar volume vary from 4.049 g/cm^3^ to 4.21 g/cm^3^ and from 43.62 cm^3^/mol to 45.43 cm^3^/mol, respectively. The density is evaluated with a measurement accuracy of 0.001, and the average absolute error is 0.051. The opposite tendency has been noticed in ρ and $\:{\text{V}}_{\text{m}}$with a fluctuation due to the incorporation of Ho_2_O_3_, having a high atomic mass, which replaces the lighter boron atoms and affects network rigidity. The presence of lead oxide also influences the structural modification. The maximum density was observed at y equals 0.1 mol% of Ho_2_O_3_. The molar volume is influenced by both the density and structural modification in the glass network. However, the reduction in density could be attributed to the production of NBOs as well as clusters of RE-ions. The impact of Ho_2_O_3_ on the glass system can also be analyzed through variations in oxygen packing density (OPD), and boron-boron distance ($\:{\text{d}}_{\text{B}-\text{B}}$). The OPD and $\:{\text{d}}_{\text{B}-\text{B}}$ vary between 55.002 $\:\text{g}.\text{a}\text{t}\text{o}\text{m}/\text{l}$ to 56.165 $\:\text{g}.\text{a}\text{t}\text{o}\text{m}/\text{l}$ and 4.690 Å and 4.749 Å. The variations in OPD, which represent the number of oxygen atoms per unit volume and $\:{\text{d}}_{\text{B}-\text{B}}$ are in compliance with the density and molar volume. As Ho_2_O_3_ content increases, the interplay between the B_2_O_3_, TeO_2_, PbO, and Na_2_O contributes to the network modification, disrupting the regular rearrangement of boron atoms and the creation of nonbridging oxygens (NBOs). This alters the OPD and $\:{d}_{B-B}$. The altering trend reflects the dynamic balance of disruption and network stability of BTPNH glasses^38,61^.

#### Interionic distance, polaron radius, and field strength

The interionic distance $\:\left({r}_{i}\right)$, polaron radius $\:\left({r}_{p}\right)$, and field strength $\:\left(F\right)$ are evaluated through the relation as follows,

The interionic distance ($\:{r}_{i}$) was evaluated through the relation^62^,

5 $$\:{r}_{i}={\left[\frac{1}{{N}_{i}}\right]}^{1/3}$$

Where, $\:{N}_{i}=\frac{y\rho\:{N}_{A}}{{M}_{av}}$, is the ion concentration, and *y* is the mol% of the dopant.

Polaron radius ($\:{r}_{p}$) was calculated by the relation^62^,

6 $$\:{r}_{p}=\frac{1}{2}{\left[\frac{\pi\:}{6{N}_{i}}\right]}^{1/3}$$

Field strength (F) was determined by the formula^23^,

7 $$\:\text{F}=\text{Z}/{\text{r}}_{\text{p}}^{2}$$

**Fig. 3:**
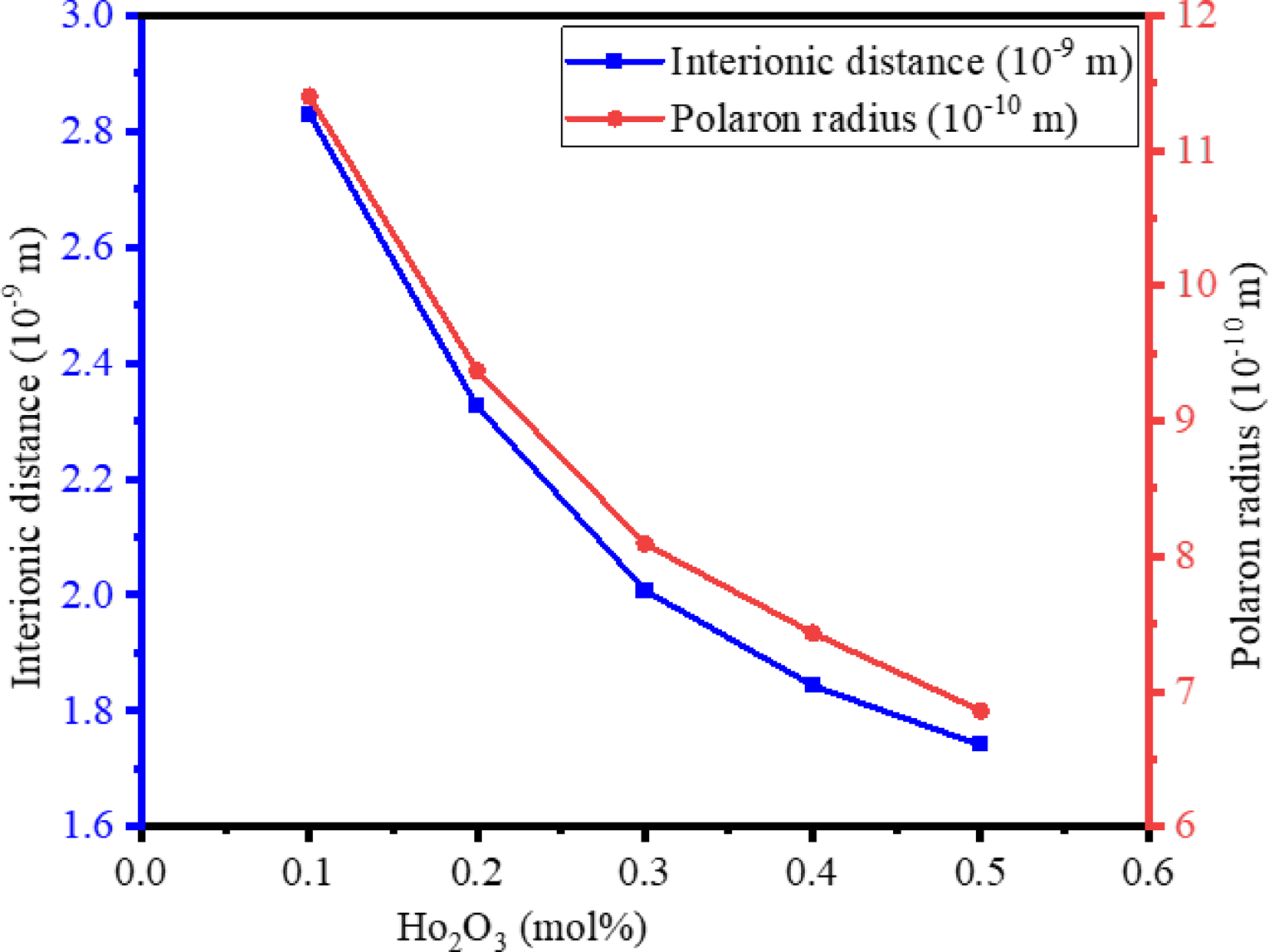
Variation of interionic distance and polaron radius with Ho_2_O_3_ in BTPNH glasses.

**Fig. 4:**
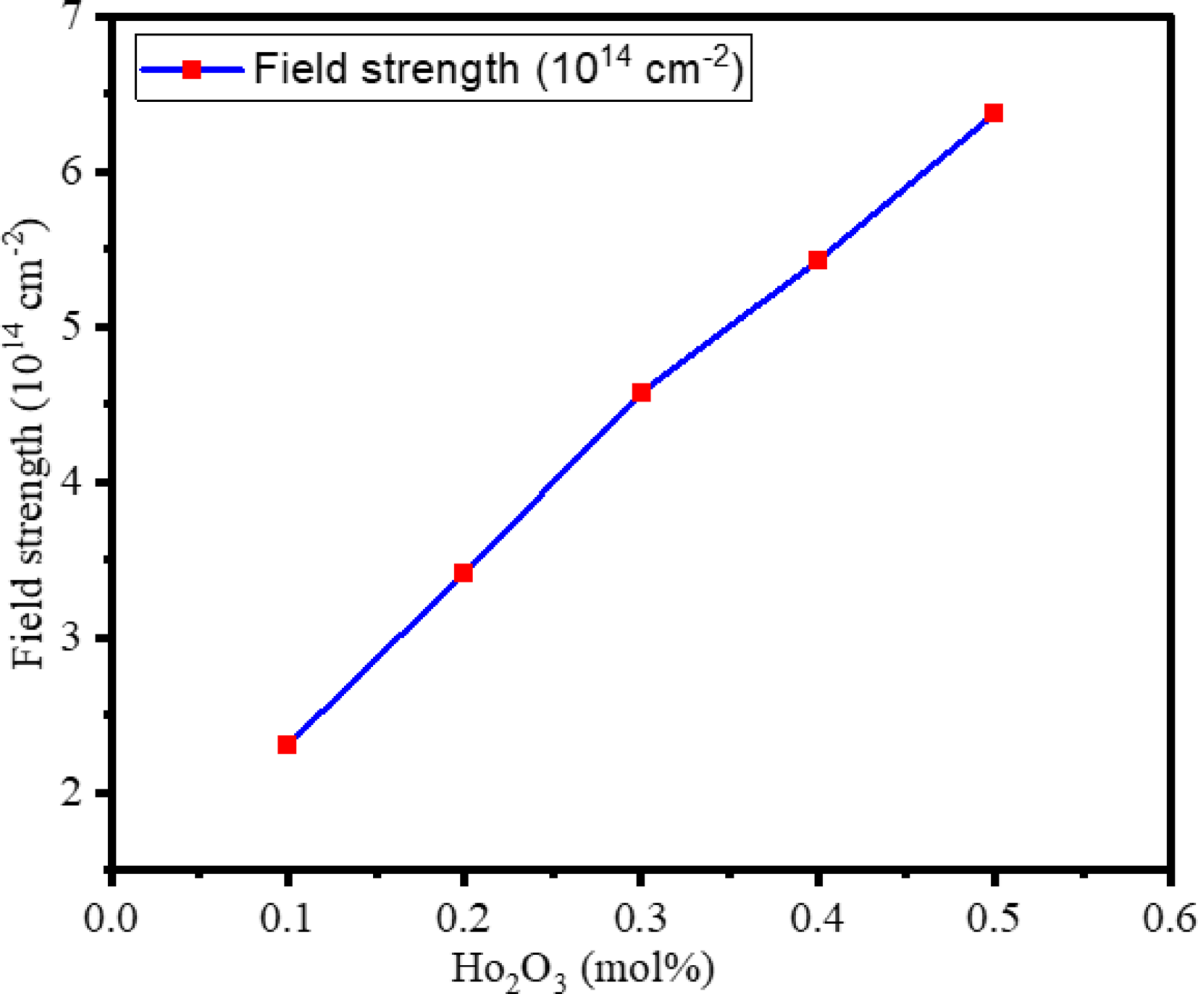
Variation of field strength with Ho_2_O_3_ in BTPNH glasses.

The variations in the interionic distance ($\:{r}_{i}$), polaron radius ($\:{r}_{p}$), and field strength (F) of BTPNH glasses are closely linked to the increasing concentration of Ho_2_O_3_. The determined values are tabulated in Table 2. The variation of interionic distance and polaron radius with Ho_2_O_3_ in BTPNH glasses is depicted in Fig. 3. Interionic distance and polaron radius vary from 2.829 nm to 1.741 nm and 11.402 Å to 6.859 Å, respectively. As the Ho_2_O_3_ concentration increases, both $\:{r}_{i}$ and $\:{r}_{p}$ tend to decrease since Ho_2_O_3_ enters the glass network, occupies the interstitial or substitutional positions, and then modifies the network, producing NBOs. The interaction between the Ho^3+^ ions charge carrier influences the polaron radius by the localization of charge carriers within a small region. The field strength (F), which measures the electric field exerted by an ion, is found to increase with Ho_2_O_3_ as shown in Fig. 4. This is because of the Ho^3+^ ion’s positive charge and small ionic radius. The addition of Ho^3+^ ions into the glass network modifies the structure, creates NBOs, and enhances the localized electric fields around the ions. Therefore, a reduction in $\:{r}_{i}$ and $\:{r}_{p}$ and an increase in field strength are attributed to the overall structural disruption, modification, and creation of NBOs with an increase in Ho^3+^ ions^6,61^.

**Table 2:** Physical parameters of BTPNH glasses^23,57,58,62,63^: density $\:\left({\uprho\:}\right)$, molar volume $\:\left({\text{V}}_{\text{m}}\right)$, Boron-Boron distance $\:\left({\text{d}}_{\text{B}-\text{B}}\right)$, ion concentration $\:\left({\text{N}}_{\text{i}}\right)$, interionic radius $\:\left({\text{r}}_{\text{i}}\right)$, Polaron radius $\:\left({\text{r}}_{\text{p}}\right)$, oxygen packing density $\:\left(\text{O}\text{P}\text{D}\right)$, and field strength (F).

Sample code		1BTPNH	2NTPNH	3BTPNH	4BTPNH	5BTPNH	6BTPNH
$\:{\uprho\:}\:\left({\text{g}\text{c}\text{m}}^{-3}\right)$	$\:\pm\:0.001$	4.135	4.212	4.049	4.193	4.067	4.147
$\:{V}_{m}\:\left({\text{c}\text{m}}^{3}/\text{m}\text{o}\text{l}\right)$	$\:\pm\:0.001$	44.370	43.621	45.431	43.905	45.359	44.543
$\:{\text{d}}_{\text{B}-\text{B}}\:$Å	$\:\pm\:0.001$	4.721	4.691	4.749	4.691	4.738	4.705
$\:{\text{N}}_{\text{i}},\:{10}^{19}\:\left(\text{i}\text{o}\text{n}\text{s}/{\text{c}\text{m}}^{3}\right)$	$\:\pm\:0.001$	-	4.415	7.953	12.344	15.931	20.279
$\:{\text{r}}_{\text{i}}\:\left(\text{n}\text{m}\right)$	$\:\pm\:0.001$	-	2.829	2.325	2.008	1.844	1.741
OPD $\:\left(\text{g}.\text{a}\text{t}\text{o}\text{m}/\text{l}\right)$	$\:\pm\:0.001$	55.216	56.165	53.927	55.801	54.012	55.002
$\:{\text{r}}_{\text{p}\:}($Å)	$\:\pm\:0.001$	-	11.402	9.371	8.094	7.434	6.859
$\:\text{F},\:{10}^{14}\:\left({\text{c}\text{m}}^{-2}\right)\:$	$\:\pm\:0.001$	-	2.307	3.416	4.579	5.428	6.375

### Structural studies

#### ATR-Fourier transform infrared spectroscopy (ATR-FTIR)

**Fig. 5:**
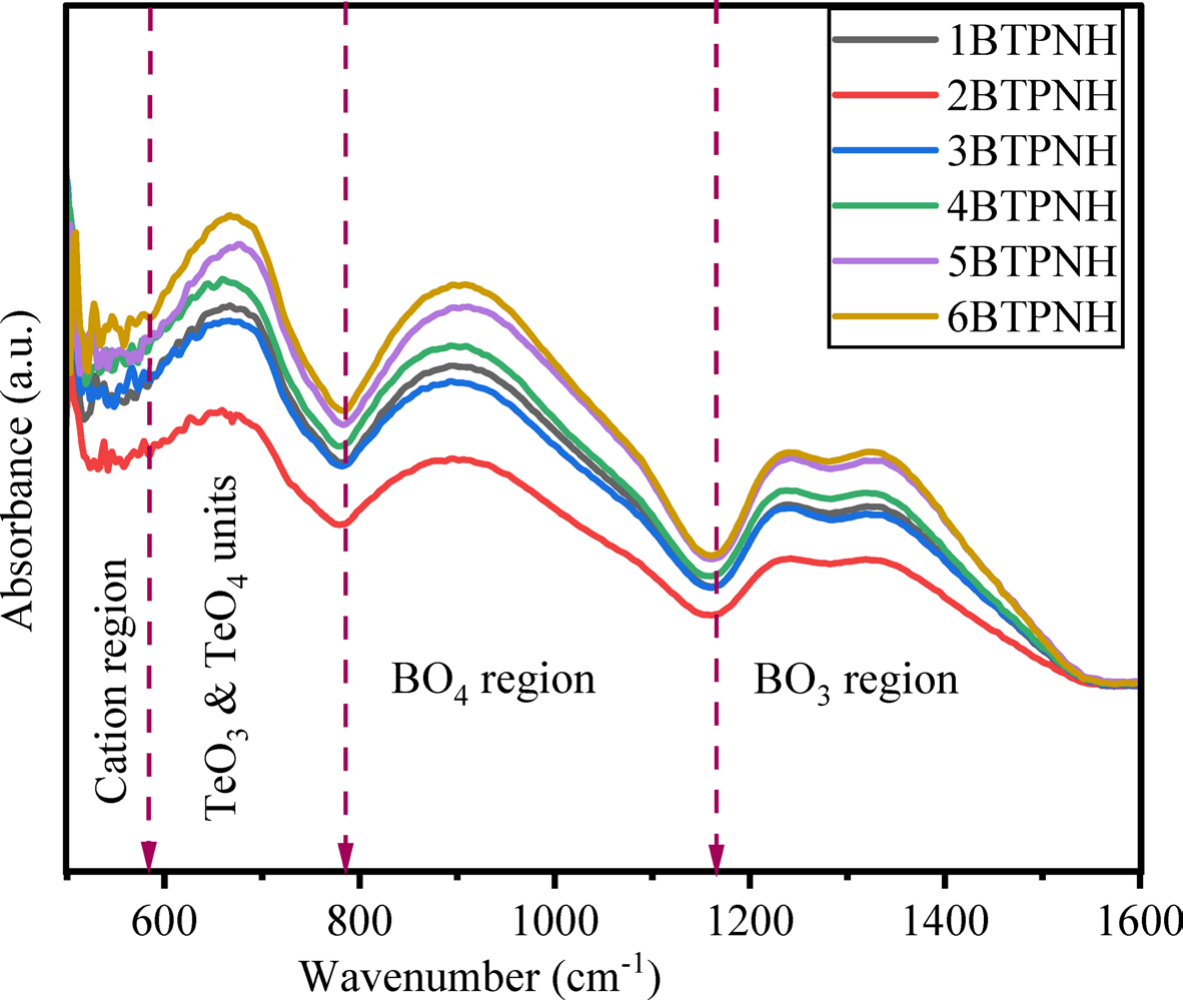
ATR-FTIR spectra of BTPNH glasses.

**Fig. 6:**
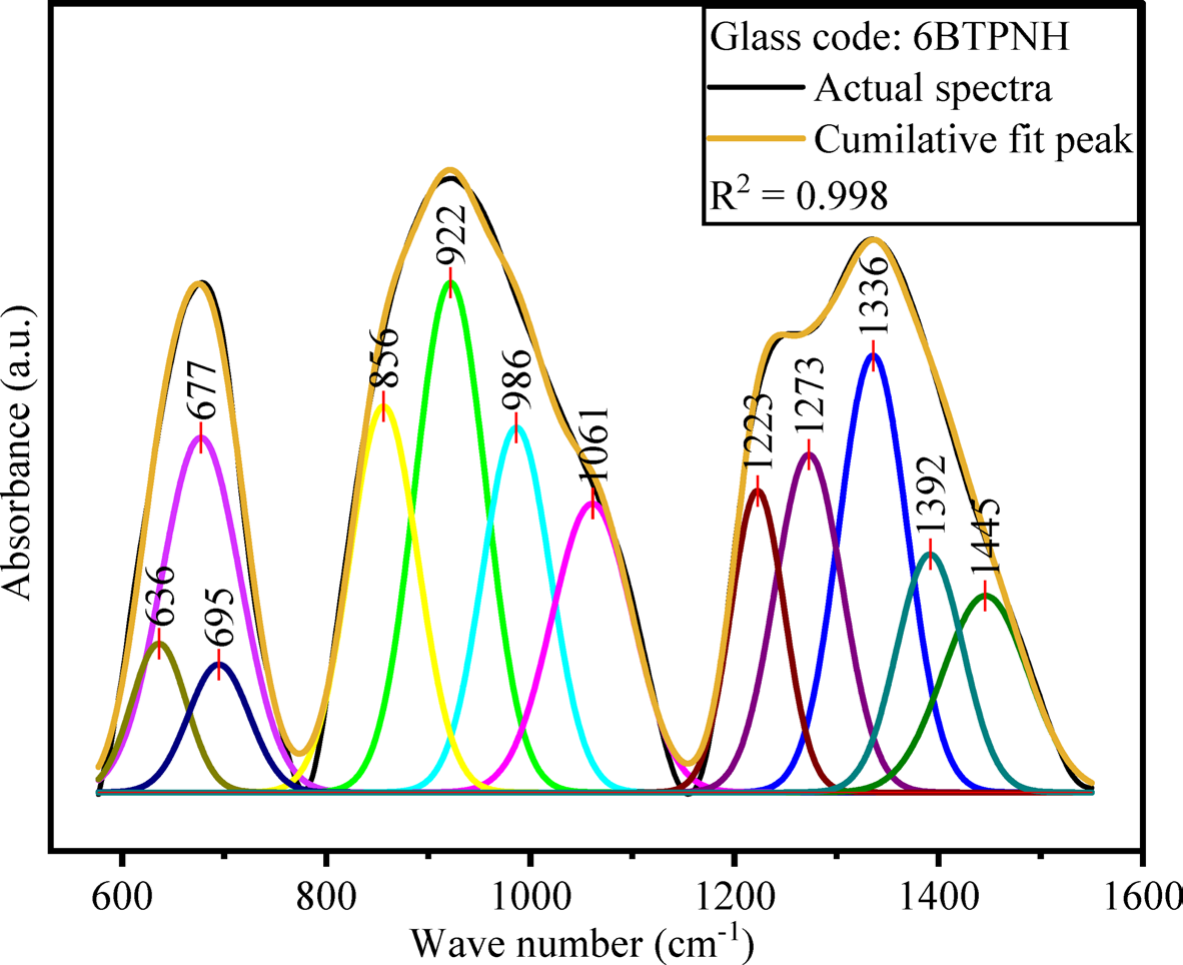
Deconvolution of the ATR-FTIR spectrum of 6BTPNH glasses.

ATR-FTIR is a highly sensitive and non-destructive technique utilised for structural analysis. The characteristic peaks that correspond to specific vibrations of functional groups within the glass network were analysed through ATR-FTIR spectroscopy. In general, for borotellurite glasses, the significant regions from 800 to 1200 cm^−1^ and 1200–1600 cm^−1^ represent the stretching and bending vibrations of BO_4_ and BO_3_ units in the borate network^64^. The region from 600 cm^−1^ − 800 cm^−1^ signifies the stretching modes of TeO_4_ and TeO_3_ units in the tellurite network. Figure 5 depicts the FTIR spectra of BTPNH glasses, which were recorded from 550 cm^−1^ to 1600 cm^−1^. Based on the literature on the borate and tellurite glass systems^16,65–67^, in BTPNH spectra, the bands around 922 cm^−1^ and 1336 cm^−1^ are linked to the BO_4_ and BO_3_ groups. It is obvious from the spectra that as the concentration of Ho_2_O_3_ increases, a change in intensity and a shift in the band are noticed. These changes are attributed to B-O stretching vibrations of BO_3_ units present in different borate configurations containing NBOs, including orthoborate, pyroborate, and metaborate groups. The spectral band around 677 cm^−1^ is designated for stretching modes of TeO_4_ and trigonal bipyramidal coordination involving bridging oxygens (BOs) as well as stretching modes of TeO_3_. At < 600 cm^−1^, peaks correspond to Pb-O links^65,68^. The FTIR band assignments for BTPNH glasses are given in Table 3. Deconvolution of FTIR spectra has been carried out by applying the peak fit tool in Gaussian Amp. Mode. A typical deconvolution spectrum (6BTPNH) is shown in Fig. 6, which reveals the peaks in the band.

The N4 parameter is defined as the fraction of 4-coordinated boron atoms (BO_4_ units) in a borate-based glass network relative to the total boron content (the sum of BO_3_ and BO_4_ units). It reflects the ratio of BO_4_ groups and probable conversion among BO_4_ and BO_3_ units. Based on the area under which the peaks associated with relevant sections of BO_4_ and BO_3_ units, N4 values were evaluated from FTIR spectra using the relation (8) given by

8 $$\:\text{N}4=\frac{\text{T}\text{o}\text{t}\text{a}\text{l}\:\text{a}\text{r}\text{e}\text{a}\:\text{u}\text{n}\text{d}\text{e}\text{r}\:{\text{B}\text{O}}_{4}}{\text{T}\text{o}\text{t}\text{a}\text{l}\:\text{a}\text{r}\text{e}\text{a}\:\text{u}\text{n}\text{d}\text{e}\text{r}\:{\text{B}\text{O}}_{3}+\text{T}\text{o}\text{t}\text{a}\text{l}\:\text{a}\text{r}\text{e}\text{a}\:\text{u}\text{n}\text{d}\text{e}\text{r}\:{\text{B}\text{O}}_{4}}$$

The evaluated N4 values corresponding to the BTPNH glasses were found to be 0.513, 0.523, 0.501, 0.515, 0.511, and 0.521. The obtained values are in compliance with the density. The higher values of N4 indicate an increased formation of BO_4_ units, resulting in higher structural stability and higher density. The lower values imply a shift towards BO_3_ units, leading to the formation of NBOs. However, the presence of TeO_2_ and PbO content promotes the BO_4_ formation, thus increasing N4 value, while Ho^3+^ doping modifies the structure, affecting the BO_4_/BO_3_ ratio.

**Table 3:** FTIR band assignments for BTPNH glasses.

Functional units	Wavenumber (cm^−1^)	Assignments	Ref
TeO_4_	631$\:-$650	TeO_4_ units with Te$\:-$O bonds.	^67,68^
	703$\:-$710	Stretching modes of [TeO_4_] tetrahedral units with BOs.	
TeO_3_	710$\:-$789	Asymmetric stretching of Te$\:=$O. Stretching vibrations of [TeO_3_] trigonal pyramidal with NBOs.	^67,69^
BO_4_	825$\:-$882	Stretching vibrations of [$\:\text{B}-\text{O}$] in BO_4_ units (tri, tetra, and pentaborate groups).	^66–68^
	908$\:-$1187	Stretching vibrations of B$\:-$O in BO_4_ units of diborate groups.	
BO_3_	1227 $\:-$ 1366	Stretching vibrations B$\:-$O in BO_3_ groups (meta, pyro, and ortho-borate groups).	^70,71^
	1373$\:-$1467	Asymmetric stretching vibrations of triangular BO_3_ units.	

### Raman studies

**Fig. 7:**
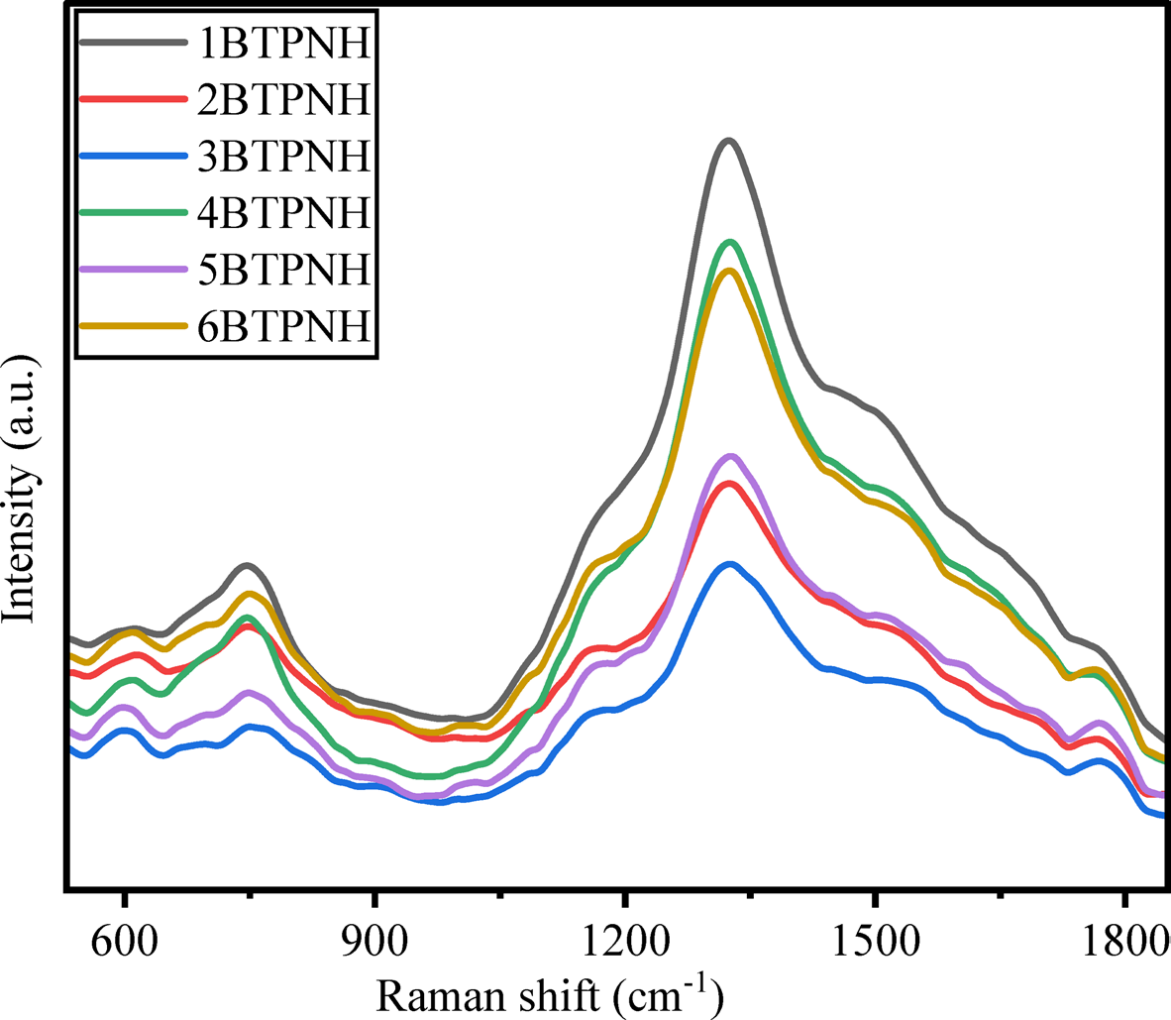
Raman spectra of BTPNH glasses.

**Fig. 8:**
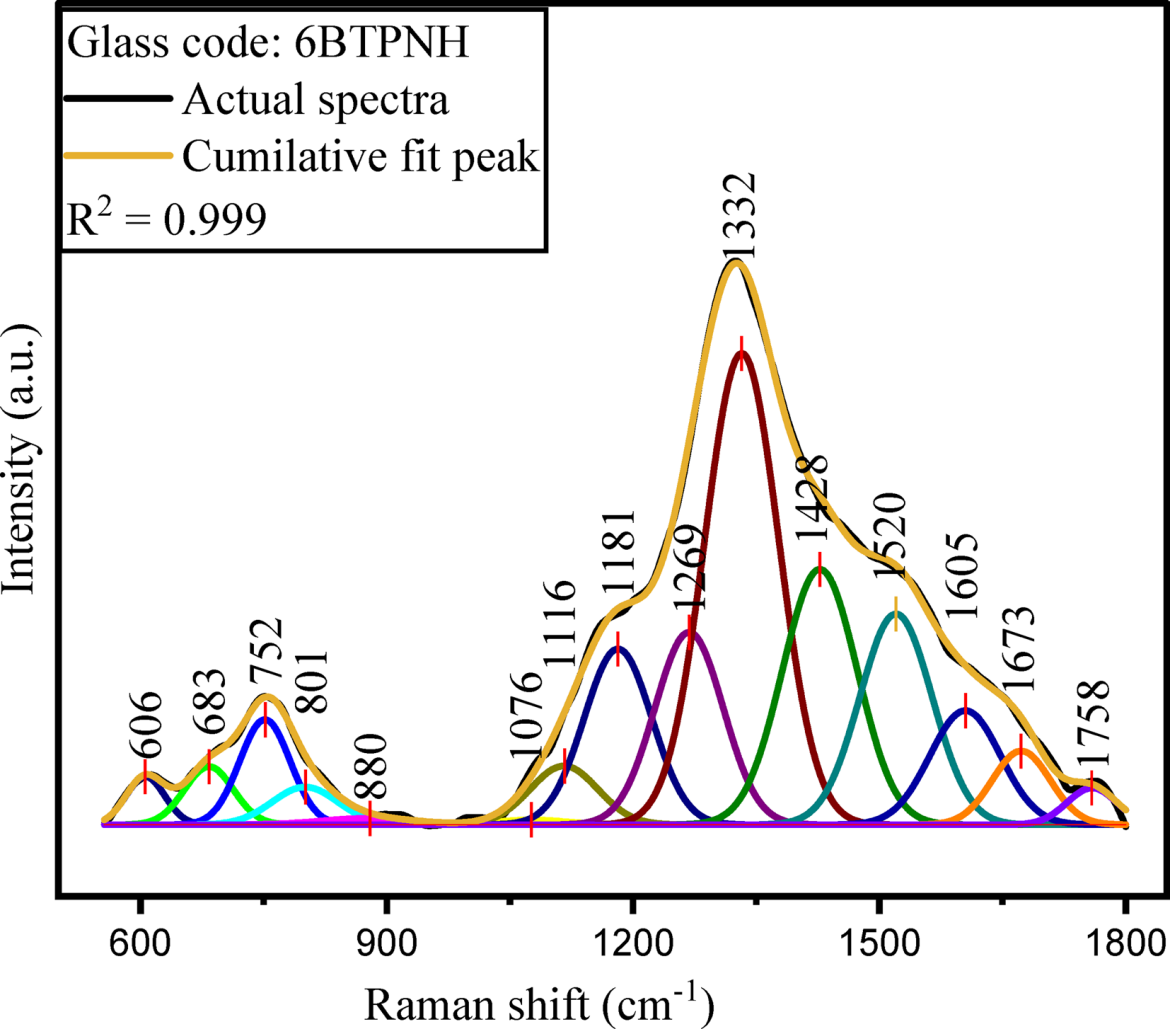
Deconvolution of Raman spectrum of 6BTPNH glasses.

Raman spectroscopy is a powerful technique to explore structural and compositional features of glass systems to ensure the appropriate suitability of materials design for various uses in optics and photonics. The Raman analysis of BTPNH glasses is done in the range of 550 cm^−1^ − 1800 cm^−1^, and corresponding spectra are shown in Fig. 7. The deconvolution of a typical spectrum (6BTPNH) of glasses is shown in Fig. 8. The Raman studies of BTPNH glasses provide insight into the glass network, including the structural changes induced by varying Ho_2_O_3_ concentrations. The boron trioxide (B_2_O_3_) content primarily contributes borate units (BO_3_ and BO_4_) to the glass network. Generally, the peaks correspond to the tetrahedral borate (BO_4_) and triangular borate (BO_3_) units that appear in the range of 800 cm^−1^ – 1200 cm^−1^ and 1200 cm^−1^ – 1600 cm^−1^^72^. The peaks for tellurite units generally appear in the range of 600 cm^−1^ – 800 cm^−1^. The boroxol ring vibrations are assigned around 800 cm^−1^. In BTPNH spectra bands in the range 877 cm^−1^ – 1200 cm^−1^ are assigned to bond vibrations in BO_4_ polyborates, which include di-, ortho-, and pyro-borates depending on BOs. The band occurred at ~ 1332 cm^−1^, is attributed to BØ_2_O^–^ triangles of BO_4_ units. The peaks appeared in the range of 1400 cm^−1^ – 1800 cm^−1^ are attributed to stretching vibrations of B-O bonds of various borate groups of B-O bonds of various borate groups and bending vibrations of O-H groups^65,73–77^. The variations in the intensity and shift in the peaks observed in the Raman spectra indicate the conversion between BO_4_ and BO_3_ units and their varying concentration, reflecting the impact of Ho_2_O_3_ content in the borate network, which results in modifying the structure, affecting the BO_4_/BO_3_ ratio. The tellurium oxide contributes to the formation of TeO_3_ and TeO_4_ structural units. Bands in the 550 cm^−1^ – 850 cm^−1^ range are associated with stretching vibrations of bonds in TeO_3_, TeO_4_, and TeO_3+δ_ units^73–75^. Modifier oxides such as PbO and Na_2_O disrupt the borate and tellurite network by breaking the bonds and forming NBOs. The Pb-O links are attributed wavelengths at < 600 cm^−1^^76^. It has been observed that as the Ho_2_O_3_ concentration increases, there is an alteration in the local environment of the borate and tellurite network, indicating modification in the glass network with changing NBOs. The bands corresponding to different borate and tellurite units of BTPNH glasses are shown in Table 4.

**Table 4:** Raman band assignments of BTPNH glasses.

Wavenumber (cm^−1^)	Assignments	Ref.
550 $\:-$ 850	Stretching vibration bonds in TeO_3_, TeO_4_, and TeO_3+δ_ units.	^4,73,74^
648 $\:-\:$700	Te$\:-$O$\:-$Te bonds with asymmetric stretching vibrations and stretching vibrations of TeO_4_ tbps.	^73,74^
773 $\:-$ 793	Continuous stretching vibrations of TeO_4_ and TeO– stretching vibrations of TeO_3+δ_ polyhedra or TeO_3_.	^73,74^
877 $\:-$ 1200	B$\:-$O bonds in BO_4_ polyborates (di-, ortho-, and pyroborates) with stretching vibrations.	^73,76,77^
1200 $\:-$ 1300	BO_3_ units with stretching vibrations of $\:\text{B}-\text{O}$ bonds.	^72,74^
1300 $\:-$ 1410	Meta-borate groups have $\:\text{B}-{\text{O}}^{-}$ bonds with stretching vibrations.	^4,73,74^
1420 $\:-$ 1605	$\:\text{B}-{\text{O}}^{-}$ bonds connected to different borate groups exhibit asymmetric stretching vibrations.	^65,73,75^
~ 1673, ~ 1758	O$\:-$H bonds with bending vibrations.	^77^

### Scanning electron microscope with energy dispersive X-ray spectroscopy

**Fig. 9:**
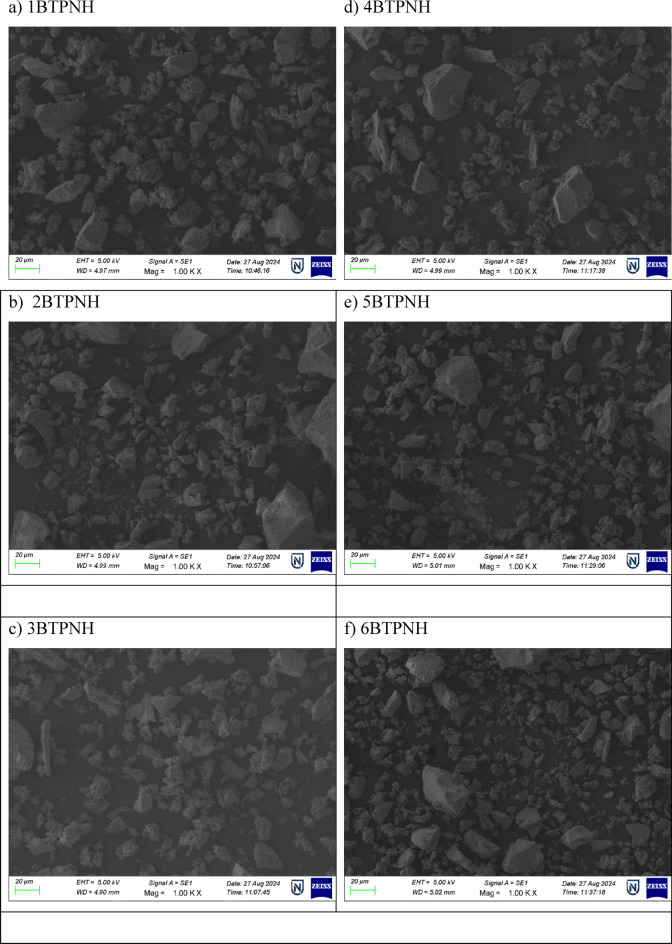
SEM images of BTPNH glasses

**Fig. 10:**
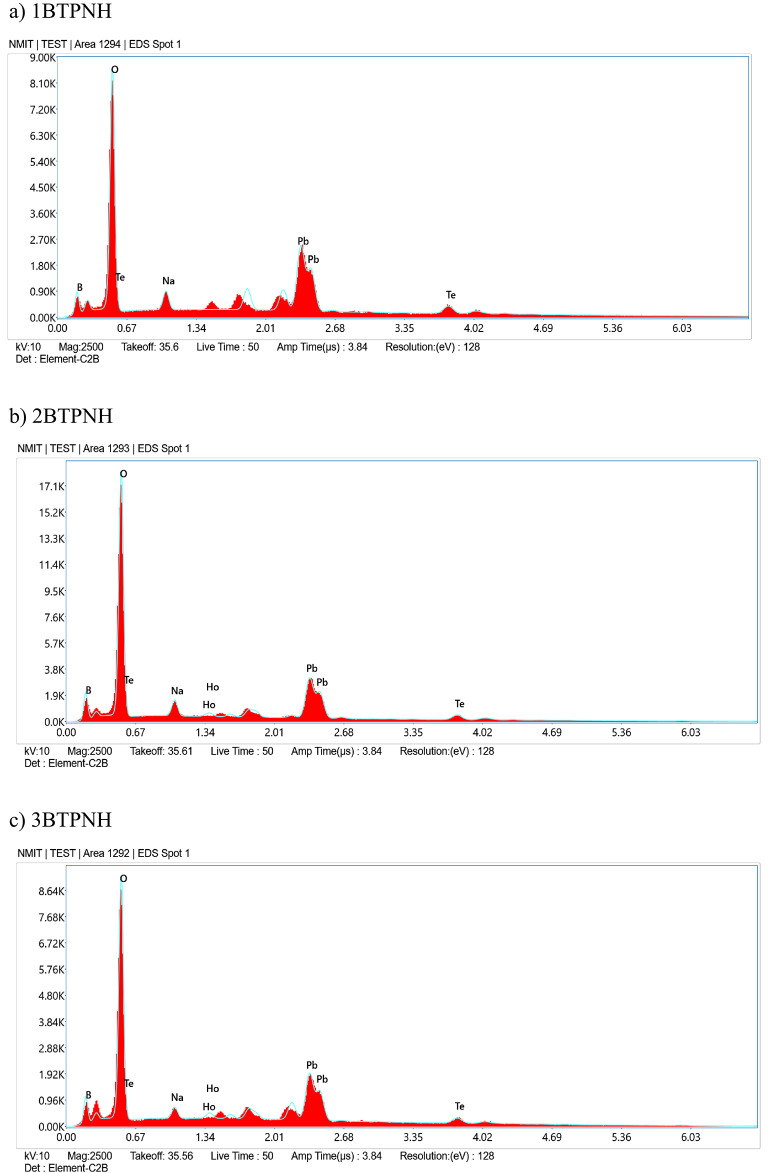

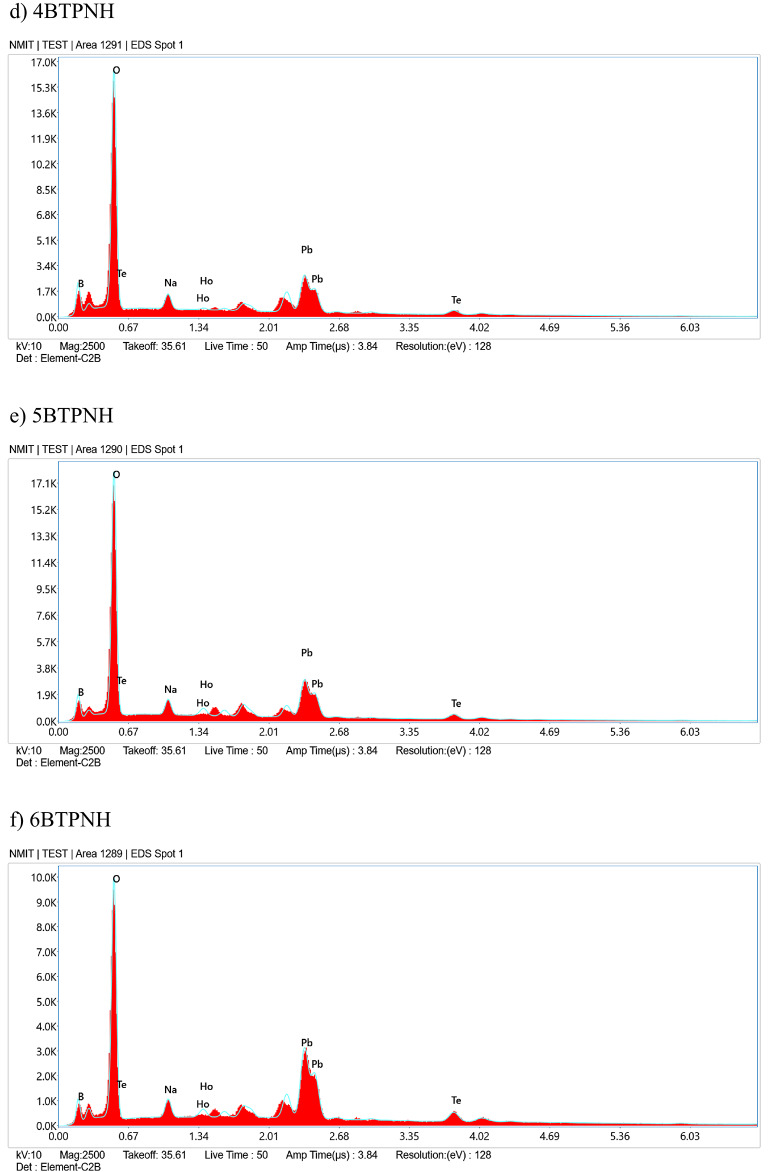
EDX spectra of BTPNH glasses.

The scanning electron microscopy-energy dispersive X-ray spectroscopy (SEM-EDX) technique was employed for examining the morphology and elemental composition of glasses. The SEM images of all the BTPNH glasses are obtained and are shown in Fig. 9. The EDX spectra of all BTPNH glasses are obtained and are depicted in Fig. 10. The SEM image with the absence of Ho^3+^ ions shows relatively smooth and homogeneous. The minor undulations in the microstructure are due to the presence of PbO and TeO_2_, which disrupt the glass by introducing NBOs. Upon addition of Ho^3+^ ions, the SEM images exhibited small granulated regions and microscale features, which could indicate the inhomogeneity and agglomeration of Ho_2_O_3_ particles, due breaking of the borate chain and the formation of NBOs, leading to uneven dispersion within the glass network. The EDX spectra provided the elemental confirmation of the composition, including boron (B), tellurium (Te), lead (Pb), sodium (Na), oxygen (O), and holmium (Ho). For 1BTPNH, the EDX spectrum displays prominent peaks corresponding to B, Te, Pb, Na, and O. However, the absence of a holmium peak confirms that the glass is holmium-free in 1BTPNH glass. The EDX spectra of holmium oxide-doped glasses showed a prominent peak corresponding to holmium along with other elements such as B, Te, Pb, Na, and O. As the incorporation of Ho^3+^ ions into the network increases, the EDX spectrum shows a relatively more prominent holmium peak. Moreover, the minor changes in the relative intensities of boron and oxygen peaks could suggest that the increase in the Ho_2_O_3_ concentration disrupts the glass network and stability by creating NBOs and altering the BO_3_/BO_4_ ratio. These observations are correlated with the physical and optical parameters of BTPNH glasses.

### Optical studies

#### UV- visible absorption spectroscopy

**Fig. 11:**
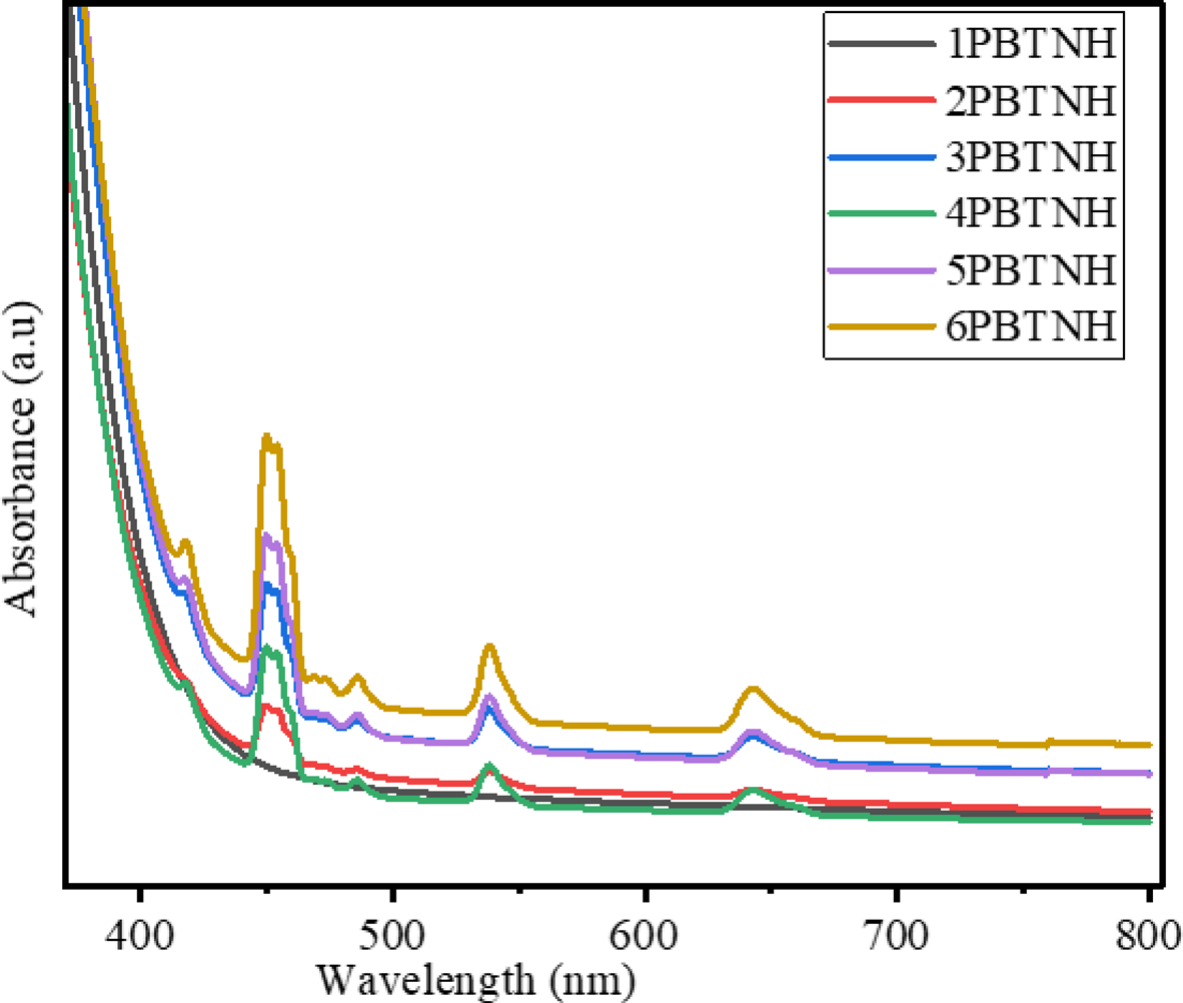
Absorption spectra of BTPNH glasses.

**Fig. 12:**
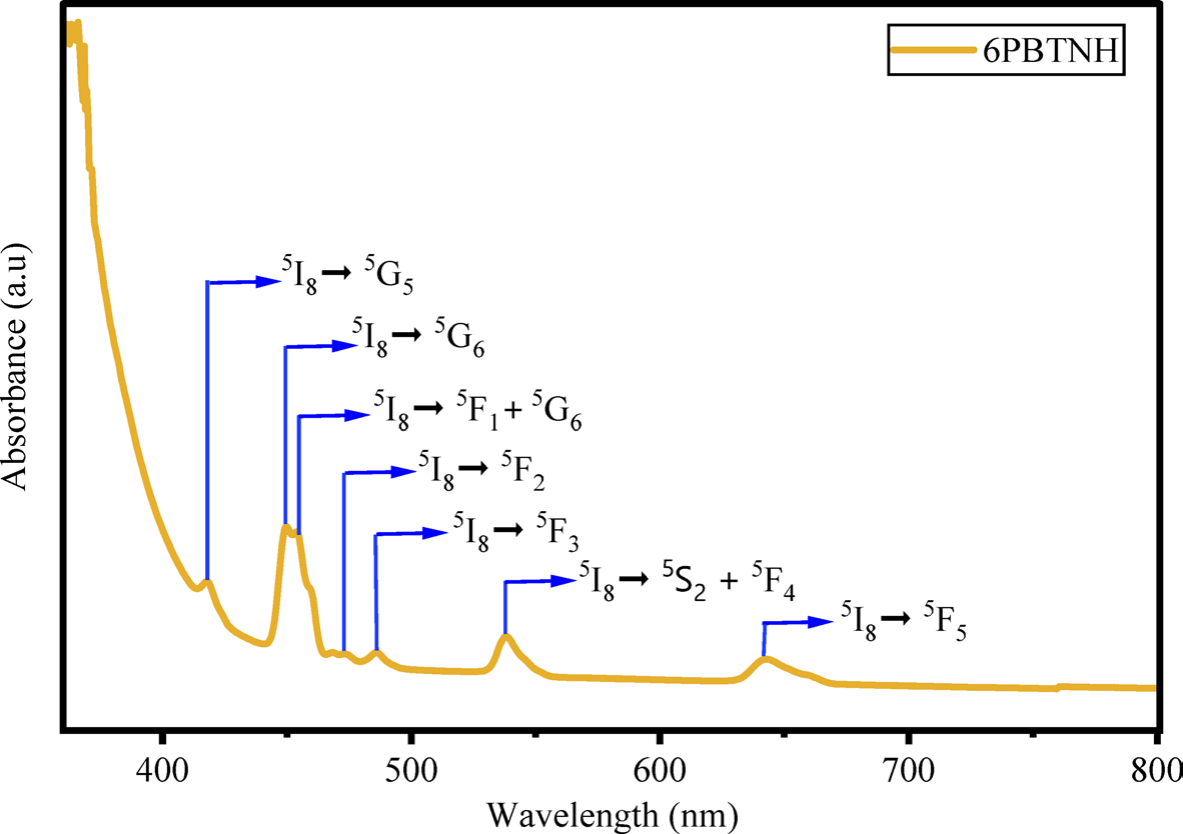
Absorption spectrum of 6BTPNH glass.

The ultraviolet spectroscopic measurements of the UV-visible absorption spectra can be used to evaluate optical properties such as optical band gap, refractive index, Urbach energy, steepness parameter, etc. The evaluated optical parameter values are listed in Table 5. The absorption spectra of BTPNH glasses were obtained in the range 350 nm to 800 nm and are shown in Fig. 11. Figure 12 shows a spectrum displaying the transition from the 5I8 state of Ho^3+^ ions to different excited states of the BTPNH glass sample. The absorption peaks occurred at 418 nm, 450 nm, 454 nm, 474 nm, 486 nm, 539 nm, and 642 nm, corresponding to the ^5^I_8_ $\:\to\:$ ^5^G_5_, ^5^I_8_ $\:\to\:$ ^5^G_6_, ^5^I_8_ $\:\to\:$ ^5^F^1^ $\:+$ ^5^G_6_, ^5^I_8_ $\:\to\:$ ^5^F_2_, ^5^I_8_ $\:\to\:$ ^5^F_3_, ^5^I_8_ $\:\to\:$ ^5^S_2_ $\:+$ ^5^F_4_, and ^5^I_8_ $\:\to\:$ ^5^F_5_ transitions^78–82^. The transitions in the wavelength range 350 nm to 800 nm were induced by electric dipole interactions. Among the observed transitions, ^5^G_6_ is a hypersensitive transition, which is particular to the circumambient atmosphere of Ho^3+^ ions, following the selection rules $\:\left|\varDelta\:\text{S}\right|=0,\:\left|\varDelta\:\text{L}\right|<2\:\text{a}\text{n}\text{d}\:\left|\varDelta\:\text{J}\right|\le\:2$. The inhomogeneity in symmetry and covalency of Ho^3+^ ions leads to hypersensitivity, potentially influencing the intensity. The absorption peaks observed at shorter wavelengths exhibit lower sensitivity due to the reduced total angular momentum associated with transitions at lower energy levels. As a result, the degree of stark splitting in these bands is comparatively smaller than that observed in transitions involving higher energy states^7,34,49^.

### Optical band gap, refractive index, urbach energy, and steepness parameter

Optical band gap refers to the direct or indirect difference in valence and conduction bands. The optical band gap energy ($\:{E}^{opt}$) is an essential optical parameter that provides a comprehensive understanding of the glass’s electronic structure and its influence on other parameters such as refractive index, Urbach energy, steepness parameters, etc. Optical band can be evaluated through the Davis–Mott equation^83^ and is given by

9 $$\:\alpha\:h\vartheta\:=B{\left[h\vartheta\:-{E}^{opt}\right]}^{m}$$

Where, $\:{\upalpha\:}\left({\uplambda\:}\right)=2.303\left[\frac{\text{A}}{\text{x}}\right]$, is the absorption coefficient. A and x are the absorbance and thickness of the glass samples, respectively. $\:h\vartheta\:$ is the photon energy, B is the band tailing parameter, and m represents the kind of transition, m $\:=$ 2, and 1/2 corresponding to the indirect allowed and direct allowed transitions, respectively.

Dimitrov and Sakka’s equation^84^ which relates to *E*_*opt*_ was utilized to determine the refractive index (n) and is given by

10 $$\:1-\sqrt{\frac{{E}_{g}}{20}}=\frac{{n}^{2}-1}{{n}^{2}+2}$$

Urbach energy (*E*_*u*_) provides the critical insights into the degree of structural disorder and localized states in the optical band gap of amorphous materials. It was evaluated using the relation^83^,

11 $$\:\text{ln}\alpha\:=\text{ln}{\alpha\:}_{0}+\left(\frac{1}{{E}_{u}}\right)h\vartheta\:$$

The steepness parameter, which provides information about the absorption edge due to the interaction of electrons with phonons, was determined using the Urbach energy through the relation^83^,

12 $$\:S={K}_{B}T/{E}_{u}$$

Where, T is the ambient temperature and $\:{K}_{B}$ is the Boltzmann constant.

**Fig. 13:**
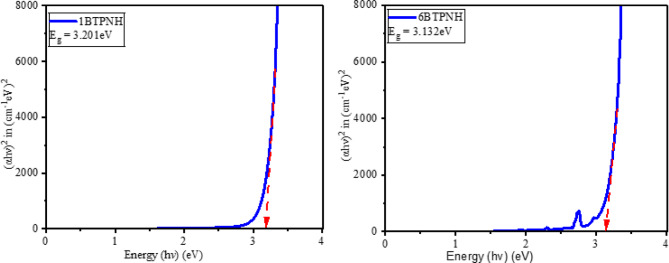
Typical plots of $\:\text{h}{\upvartheta\:}\:\text{v}\text{s}\:{\left[{\upalpha\:}\text{h}{\upvartheta\:}\right]}^{2}$ of BTPNH glasses.

**Fig. 14:**
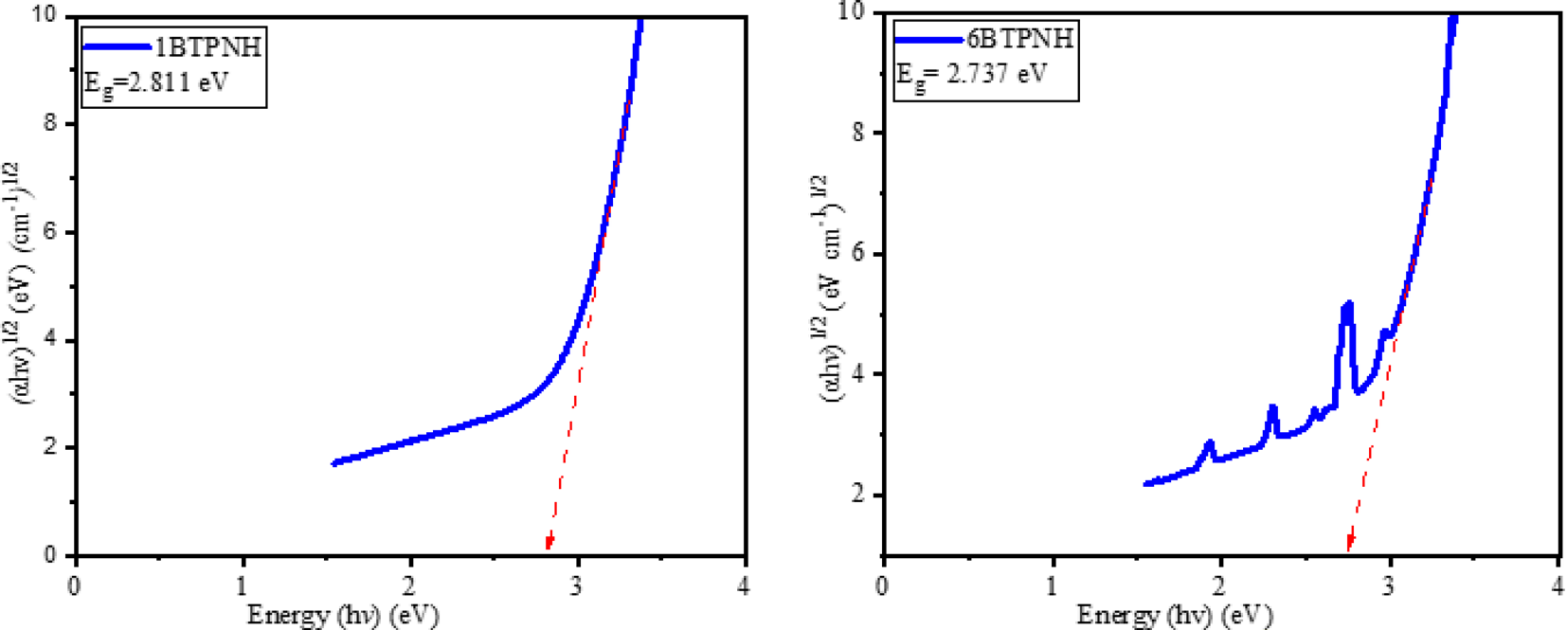
Typical plots of $\:\text{h}{\upvartheta\:}\:\text{v}\text{s}\:{\left[{\upalpha\:}\text{h}{\upvartheta\:}\right]}^{1/2}$ of BTPNH glasses

**Fig. 15:**
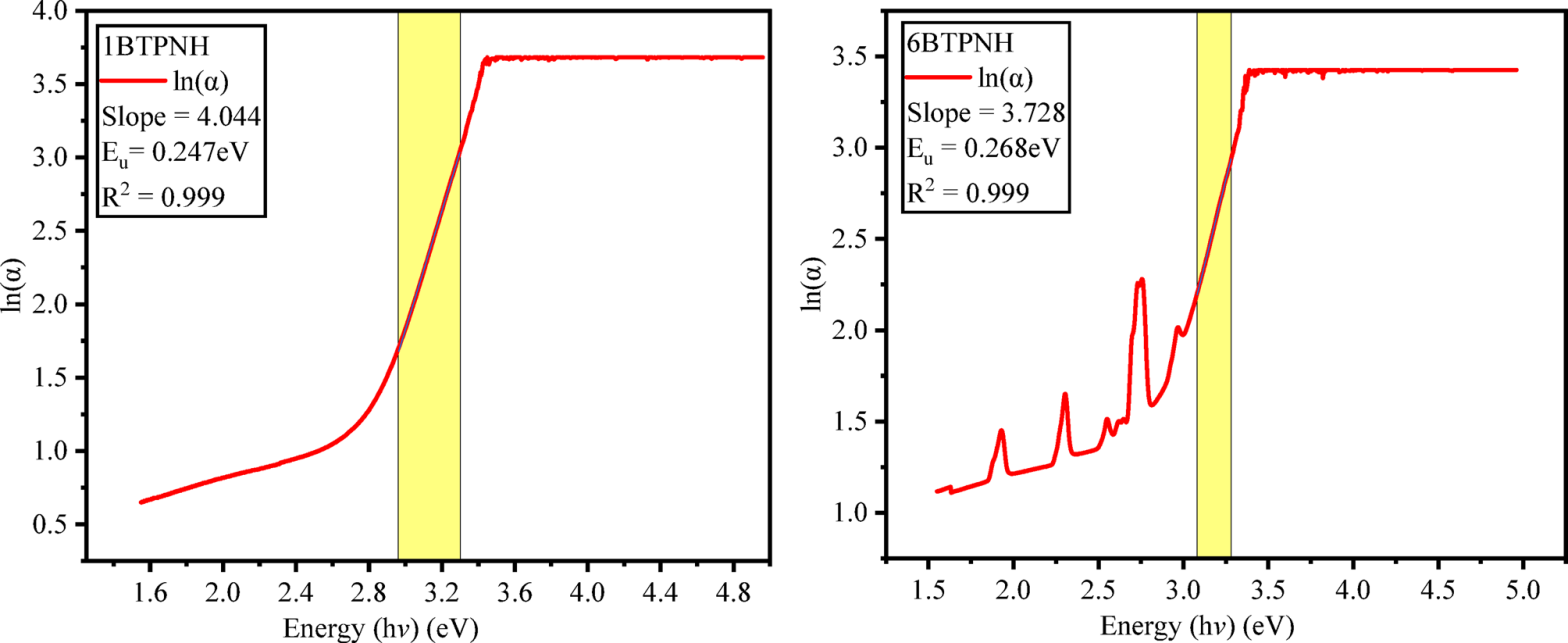
Typical plots of hϑ vs ln ⁡α of BTPNH glasses.

Tauc’s plots were employed to determine the optical band gaps. Figure 13 and Fig. 14 represent typical Tauc’s plots of direct and indirect band gap energies. The direct and indirect band gap energy values of BTPNH glasses are listed in Table 5. They were found in the range of 3.115 eV to 3.201 eV and 2.701 eV to 2.811 eV, respectively. The incorporation of Ho^3+^ ions into the host matrix disrupted the glass network by breaking the BOs. This causes the creation of NBOs, which introduce the localized defect states within the band gap. These states lower the effective band gap energy by creating intermediate energy levels for electron transitions. There is a slight band gap shift since density varies non-linearly due to borate network reorganization (BO_3_↔BO_4_). In addition, the changes in the polaron radius and inter-ionic distance also influence enhancing the electron localization, which leads to changes in band gap energy^38,85,86^. The refractive values were found between 2.345 and 2.367. A sharp increase in the refractive index is observed from 0.0 mol% to 0.1 mol% due to the introduction of Ho^3+^ ions, which have a larger ionic radius and high polarizability. Then the refractive index shows minor variations because of the structural modification due to a change in NBO and BO_4_/BO_3_ ratio, and possible formation of Ho^3+^ ion clusters, reducing the effective polarizability. The variation of refractive index is in correlation with the optical band gaps. As the Ho^3+^ ions doping increases, the optical band gap varies via localized 4f-4f transitions contributing to changes in refractive index^38,80,87^. Urbach energy and steepness parameter values of BTPNH glasses are listed in Table 5. Urbach energy varies from 0.247 eV to 0.269 eV. The typical plots of hν versus $\:\text{ln}\alpha\:$ are shown in Fig. 15. The range of *Eu* values indicates small-scale structural disorder, such as Ho^3+^ incorporation into the host matrix. The lower *Eu* values (< 0.3 eV) imply reduced tail states near band edges, improving transparency and optical homogeneity. Also, the minor variations could reflect the measurement sensitivity to localized defects^38^. The steepness parameter values of BTPNH glasses vary from 0.105 to 0.096. Initially, as the Ho^3+^ ions are incorporated, the steepness parameter drops, indicating disruption of the network, which leads to structural disorder and more localized states. As Ho^3+^ increases, the steepness parameter is found to increase and vary slightly, demonstrating an improved structural stability. However, there is a competing effect of network disruption and stabilization at 0.5 mol% of Ho^3+^ ions^23,36,88^.

### Molar refractivity, metallization criterion, and optical dielectric constant

The Lorentz – Lorentz equation^84^ is used to evaluate the molar refractivity ($\:{R}_{m}$) and is given by

13 $$\:{R}_{m}={V}_{m}\left(\frac{{n}^{2}-1}{{n}^{2}+2}\right)$$

The metallization criterion $\:\left({M}_{c}\right)$ and optical dielectric constant ($\:{\varepsilon}^{opt}$) are evaluated by the relations^84^,

14 $$\:{M}_{c}=1-\frac{{V}_{m}}{{R}_{m}}$$

15 $$\:{\epsilon}^{opt}={n}^{2}-1$$

**Fig. 16:**
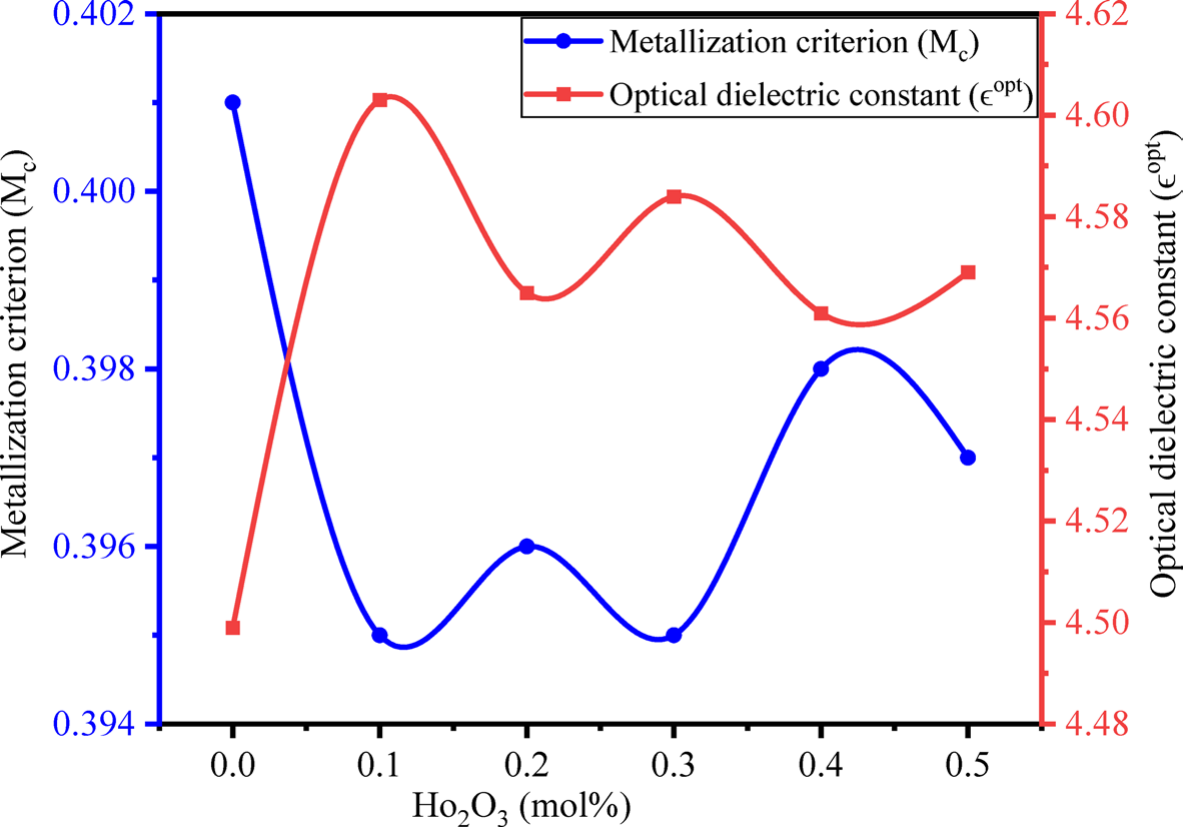
Variation of M_c and $\epsilon^{opt}$ with Ho_2_O_3_(mol%) in BTPNH glasses .

The calculated values $\:{R}_{m}$, $\:{M}_{c}$ and $\:{\epsilon}^{opt}$ are tabulated in Table 5. The total polarizability of a mole in a material can be expressed through the molar refractivity. It also helps in evaluating the molar electronic polarizability, which signifies how a cloud of electrons responds to an electric field. Molar refractivity and molar electronic polarizability are directly proportional. Initially, molar refractivity drops as the introduction of Ho^3+^ drops due to structural reorganization in the matrix and increased network cross-linking, slightly reducing the effective polarizability per mole. As the Ho^3+^ concentration increases, there is a slight increment and decrement in the molar refractivity due to the creation of NBOs and the larger ionic radius of HMOs and RE-ions^74,89^. Another key role of the Lorentz-Lorentz relation is an estimation of the metallic nature of solids by considering the ratio of *R*_*m*_ and *V*_*m*_. In accordance with the Herzfeld theory of metallization, the material shows insulating behavior if $\:\frac{{R}_{m}}{{V}_{m}}<1$ and metallic behavior if $\:\frac{{R}_{m}}{{V}_{m}}>1$. Based on these criteria, the BTPNH glasses show an insulating nature. The M_c_ values of BTPNH glasses are in the range of 0.395–0.401, and the variation of the metallization criterion (M_c_) is shown in Fig. 16. As per Wang et al. the oxides with an M_c_ range of 0.35–0.45 are suitable for optical devices^80^. The optical dielectric constant ($\:{\epsilon}^{opt}$) values are in the range of 4.499 to 4.603. The $\:{\epsilon}^{opt}$ variations are depicted in Fig. 16. The variation in the $\:{\epsilon}^{opt}$ arises from the structural modification induced by Ho^3+^ doping, primarily through changes in the NBOs concentration and electronic polarizability^84^.

### Numerical aperture, reflection loss, and transmission

The numerical aperture (NA), reflection loss ($\:{R}_{L}$), and transmission ($\:T$) of BTPNH glasses are determined by the relations^90^.

16 $$\:NA\hspace{0.17em}=\hspace{0.17em}n\sqrt{2\varDelta\:}$$

17 $$\:{R}_{L}={\left(\frac{n-1}{n+1}\right)}^{2}$$

18 $$\:T=\frac{2n}{{n}^{2}+1}$$

Where, n and $\:\varDelta\:$ be the refractive index of glasses and a small change in refractive index (0.01).

The NA is defined as a measure of its light-gathering and guiding capability and is a function of the refractive index. The obtained values of NA are mentioned in Table 5. The NA values are in the range of 0.332–0.335. These variations are due to changes in n and demonstrate that the glass system holds optical uniformity and functionality for optical applications. The reflectance and transmission values are in the range of 16.2% − 16.5% and 71.6% − 72.2% respectively. The very consistent values show that the glass host can accommodate Ho^3+^ ions without significant optical loss.

### Electronic oxide polarizability, optical basicity, and electronegativity

Electronic oxide polarizability ($\:{\alpha\:}_{{o}^{2-}}$) refers to the capacity of oxide ions, (O^2–^), within a glass matrix to undergo polarization in response to an applied electric field. It is evaluated through the relation^49^,

19 $$\:{\alpha\:}_{{o}^{2-}}=\frac{\left[\left(\frac{{V}_{m}}{2.52}\right)\left(\frac{{n}^{2}-1}{{n}^{2}+2}\right)-\sum\:{\alpha\:}_{cation}\right]}{{N}_{{o}^{2-}}}$$

Where, $\:{\alpha\:}_{cation}$ is the molar cation polarizability, n is the refractive index, $\:{N}_{{o}^{2-}}=$ Number of oxide ions in glass, *V*_*m*_ is the molar volume.

Optical basicity (Λ) and electronegativity ($\:{\chi\:}_{e}$) are estimated by the relations^49^,

20 $$\:\varLambda\:=1.67\left[1-\frac{1}{{\alpha\:}_{{o}^{2-}}}\right]$$

21 $$\:{\chi\:}_{e}=\frac{\varLambda\:}{0.75}+0.25$$

**Fig. 17:**
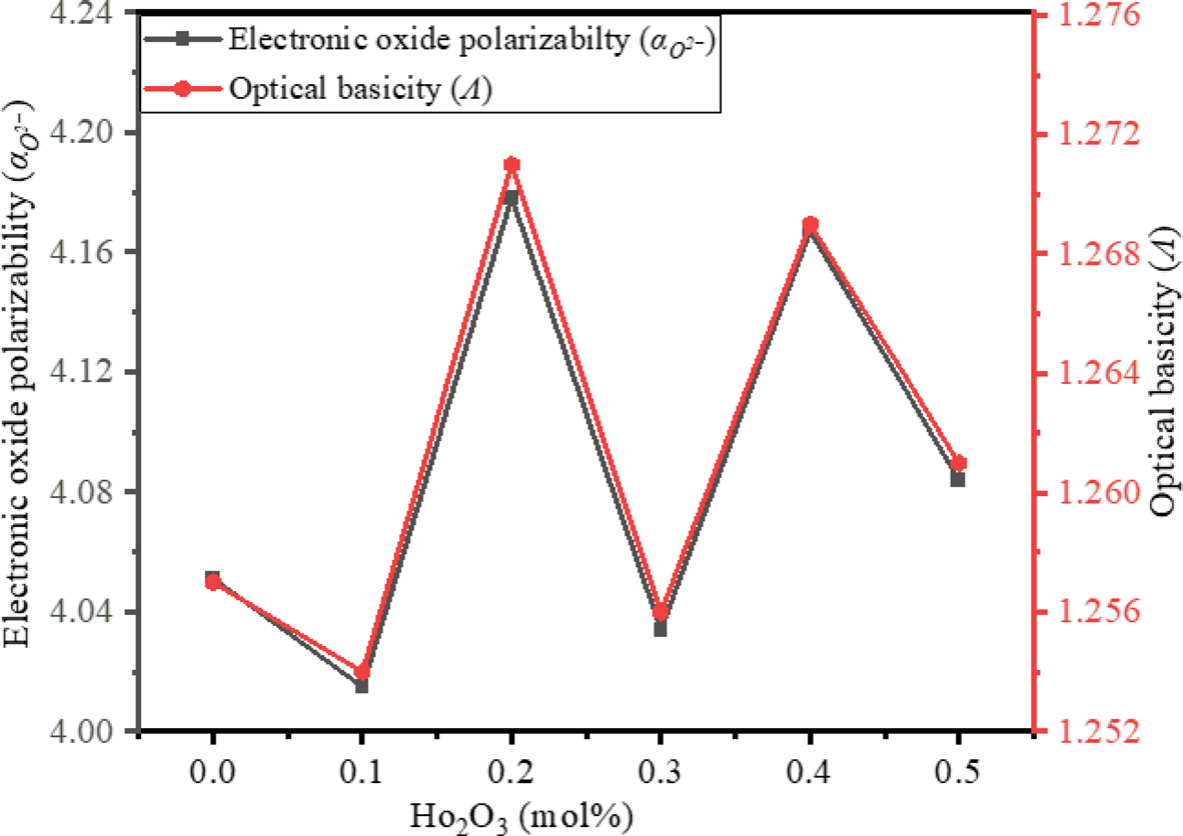
Variation of $\alpha_{o^{2}}$α_(o^(2-) ) and Λ with Ho2O3 (mol%) in BTPNH glasses.

$\:{\alpha\:}_{{o}^{2-}}$ is evaluated by considering the molar polarizability values $\:{\propto\:}_{B}=0.002$, $\:{\propto\:}_{Te}=1.595$, $\:{\propto\:}_{Pb}=3.623$, $\:{\propto\:}_{Na}=0.181$, and $\:{\propto\:}_{Ho}=0.91$^49,91,92^. The evaluated values of $\:{\alpha\:}_{{o}^{2-}}$, *Λ*, and $\:{\chi\:}_{e}$ are tabulated in Table 5. $\:{\alpha\:}_{{o}^{2-}}$ values found between 4.015 and 4.178 . The variations of $\:{\alpha\:}_{{o}^{2-}}$ in BTPNH glasses are due to structural modifications resulting from the interconversion of borate units. The decrement in the $\:{\alpha\:}_{{o}^{2-}}$ is attributed to the conversion of BO_4_ to BO_3_ and the creation of NBOs, leading to less compactness in the glass system. The increment in the $\:{\alpha\:}_{{o}^{2-}}$ is ascribed to the conversion of BO_3_ to BO_4_ units, leading to more compactness in the glass system^34^. The acid-base nature of BTPNH glasses can be expressed through optical basicity (*Λ*). It provides insight into oxygen’s electron donor ability in the glass system. The *Λ* values of BTPNH glasses are in the range from 1.254 to 1.271. The increase or decrease in *Λ* can be ascribed to the substitution of less basic B_2_O_3_ by more basic Ho_2_O_3_ and the presence of high basicity modifier oxides (TeO_2_ and PbO). In addition, the variation in *Λ* is also due to changes in the covalency in cation-oxygen bonding. The increment in the *Λ* is due to an increment in the negative charges on the O-atoms, which leads to an increment in the covalency in cation–oxygen bonding and vice versa^84,91^. Figure 17 depicts the variation of $\:{\alpha\:}_{{o}^{2-}}$ and $\:\varLambda\:$ with Ho_2_O_3_ (mol%) in BTPNH glasses. Another important parameter, electronegativity ($\:{\chi\:}_{e}$) is determined by using Eq. (21), and the values are listed in Table 5. Electronegativity in glasses indicates the tendency of atoms in the glass network to attract electrons. It affects the bonding nature in the glass network. The $\:{\chi\:}_{e}$ values are in the range of 1.922 to 1.945. As Ho^3+^ ions are introduced into the host matrix, slight fluctuations are observed in the $\:{\chi\:}_{e}$. These variations in $\:{\chi\:}_{e}$ are attributed to the reorganization within the glass matrix due to changes in the relative concentration of NBOs and BOs and alterations in the bonding environment. Higher values indicate a slightly stronger electron-attracting tendency and vice versa^23,38,93^.

**Table 5:** Optical parameters of BTPNH glasses^49,83,92,94^: indirect band gap energy $\:\left({E}_{g}^{ind}\right)$, direct band gap energy $\:\left({E}_{g}^{d}\right)$, Urbach energy $\:\left(\varDelta\:E\right)$, steepness parameters $\:\left(S\right)$, refractive index $\:\left(n\right)$, numerical aperture $\:\left(NA\right)$, optical dielectric constant $\:\left({\epsilon}^{opt}\right)$, electrical susceptibility $\:\left(\:\chi\:\right)$, molar refraction $\:\left({R}_{m}\right)$, molar polarizability $\:\left({\alpha\:}_{m}\right)$, reflection loss $\:\left({R}_{L}\right)$, transmission coefficient $\:\left(T\right)$, metallization criterion $\:\left({M}_{c}\right)$, electronic oxide polarizability $\:\left({\alpha\:}_{{o}^{2-}}\right)$, optical basicity $\:\left(\varLambda\:\right)$, electronegativity $\:\left({\chi\:}_{e}\right).$.

Sample code	1BTPNH	2NTPNH	3BTPNH	4BTPNH	5BTPNH	6BTPNH
	$\:\pm\:0.001$	$\:\pm\:0.001$	$\:\pm\:0.001$	$\:\pm\:0.001$	$\:\pm\:0.001$	$\:\pm\:0.001$
$\:{E}_{g}^{d}\left(eV\right)$	3.201	3.115	3.144	3.130	3.150	3.132
$\:{E}_{g}^{ind}\left(eV\right)$	2.811	2.701	2.716	2.703	2.779	2.737
$\:{E}_{u}\:\left(eV\right)$	0.247	0.269	0.263	0.248	0.251	0.268
S	0.105	0.096	0.098	0.104	0.103	0.096
n	2.345	2.367	2.359	2.363	2.358	2.362
$\:{R}_{m}$	26.621	26.408	27.414	26.536	27.361	26.857
$\:{\alpha\:}_{m}$	10.563	10.479	10.878	10.531	10.857	10.657
$\:{M}_{c}$	0.401	0.395	0.396	0.395	0.398	0.397
$\:{\epsilon}^{opt}$	4.499	4.603	4.565	4.584	4.561	4.579
$\:NA$	0.332	0.335	0.334	0.335	0.333	0.334
$\:\chi\:$	0.358	0.366	0.363	0.365	0.363	0.365
$\:{R}_{L}$	0.162	0.165	0.164	0.165	0.163	0.164
T	0.722	0.716	0.718	0.717	0.719	0.718
$\:{\alpha\:}_{{o}^{2-}}$Å	4.051	4.015	4.178	4.034	4.167	4.084
Λ	1.257	1.254	1.271	1.256	1.269	1.261
$\:{\chi\:}_{e}$	1.926	1.922	1.945	1.925	1.942	1.932

### Photoluminescence studies

#### Emission spectra

Ho^3+^-ions in the borotellurite glass systems occupy interstitial sites surrounded by oxygen atoms of B_2_O_3_ and TeO_2_ with primarily ionic interactions. Network modifiers like PbO and Na_2_O increase network flexibility, creating a distorted, asymmetric coordination environment with a coordination number of 6 to 8. The low phonon energy (due to TeO_2_) minimizes the non-radiative losses, enabling 4f-4f radiative transition in the Ho^3+^ ions, such as ^5^f_5_ to ^5^I_8_ transitions in the visible region^33,80,95^.

**Fig. 18:**
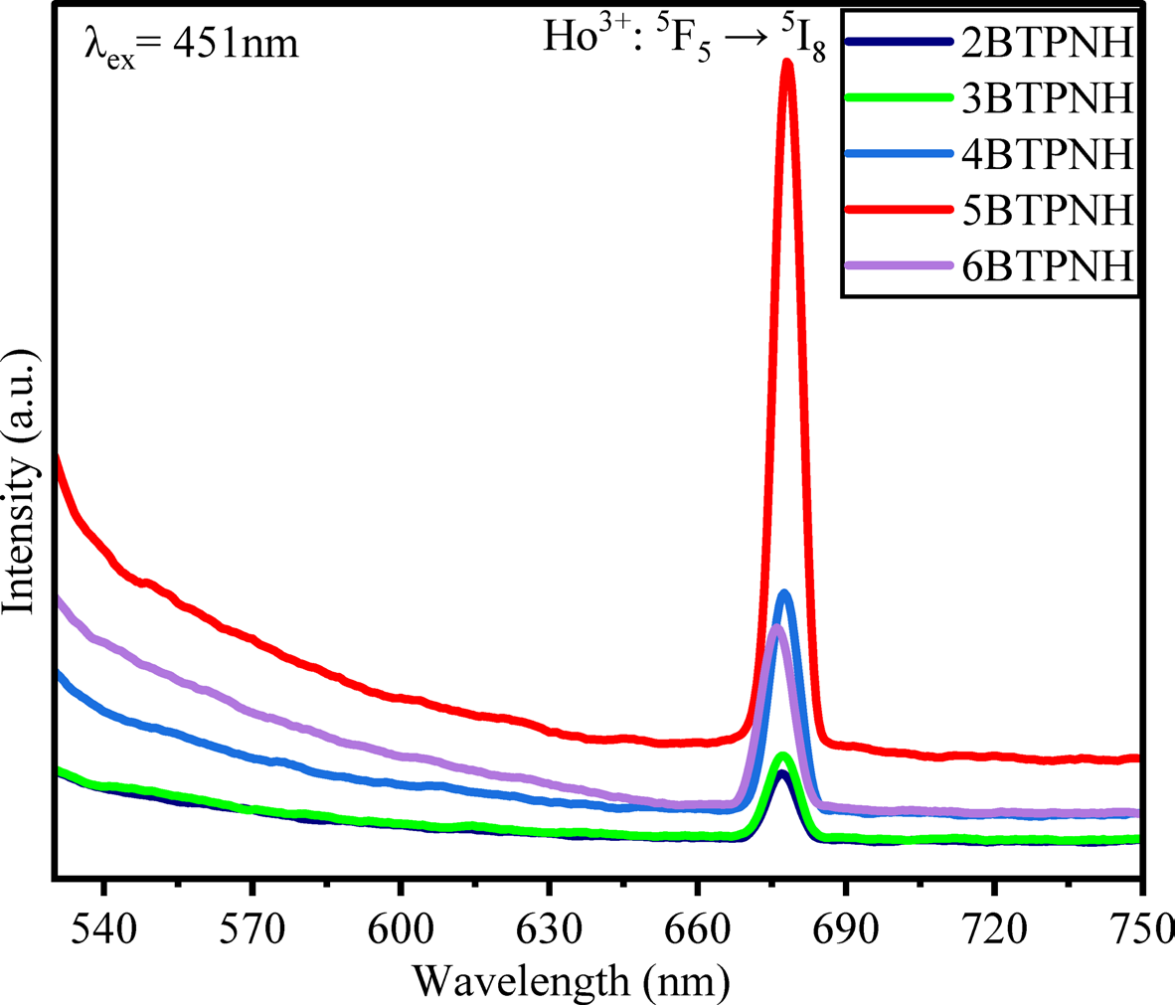
Emission spectra of BTPNH glasses.

**Fig. 19:**
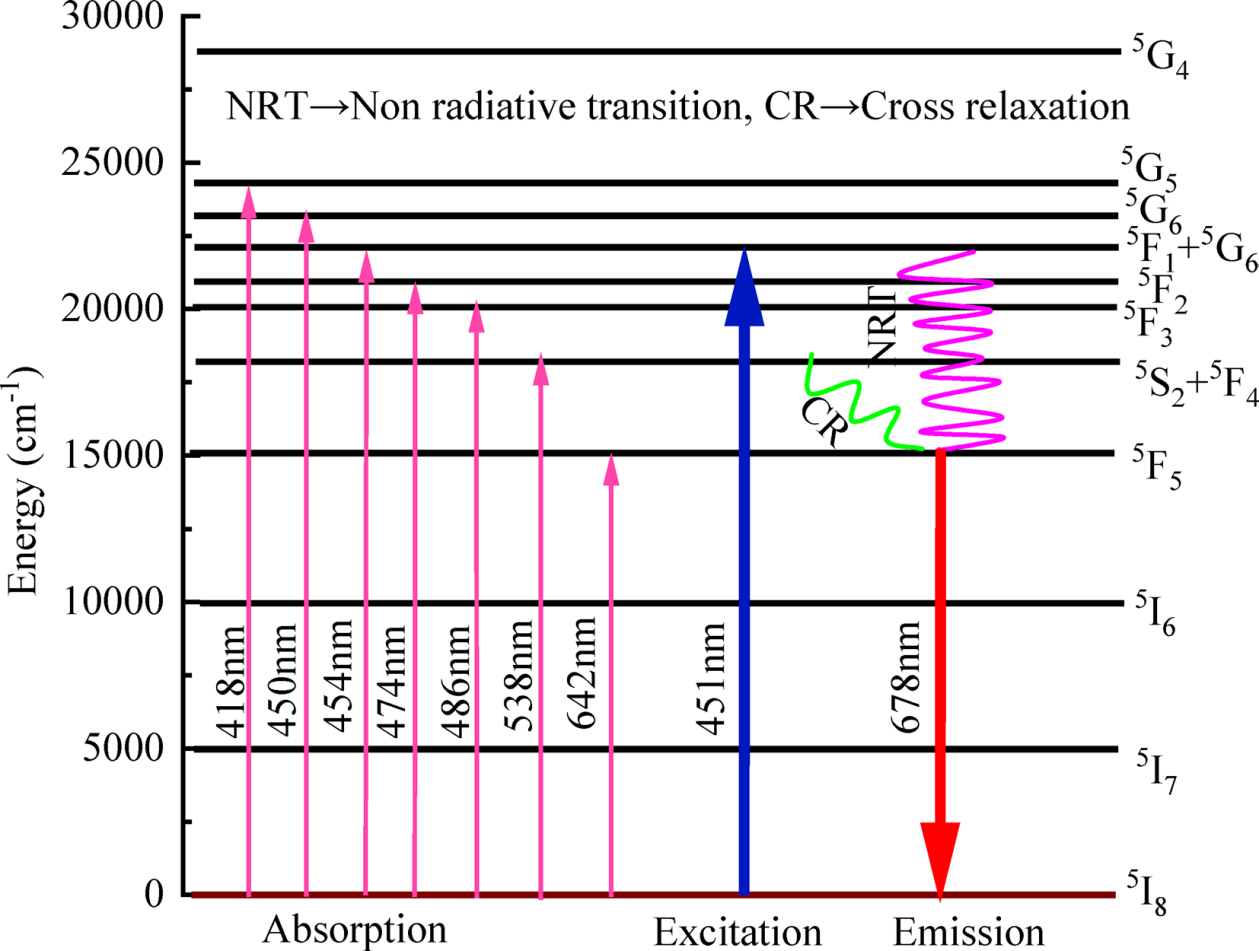
Energy level diagram: Ho_2_O_3_ + B_2_O_3_ + TeO_2_ + PbO + Na_2_O glasses.

The Photoluminescence (PL) of prepared BTPNH glasses is recorded in the range 500 nm to 750 nm, at the excitation wavelength 451 nm. Emission spectra and energy level diagrams for BTPNH glasses are shown in Fig. 18 and Fig. 19. It exhibits a sharp, strong red emission at 678 nm due to the 4f – 4f electronic transitions of Ho^3+^ ions, specifically the ^5^F_5_ → ^5^I_8_ of holmium ions. These transitions are shielded by outer 5 s and 5p orbitals, reducing sensitivity to the glass matrix and producing narrow, well-defined emission lines. The strong red emission is primarily attributed to cross relaxation processes, which provide redistribution of population from higher states (^5^S_2_) to lower states (^5^F_5_), driven by dipole-dipole interaction between the ions, the host’s low phonon energy environment, minimizing non-radiative energy losses. Additionally, the PbO$\:-$B_2_O_3_$\:-$TeO_2_ glass matrix enhances the emission intensity due to its high refractive index, which amplifies the local electric field around Ho^3+^ ions. This effect increases radiative transition probabilities, resulting in stronger luminescence^35,96–98^. The intensity of emission bands is increased up to 0.4 mol% (5BTPNH) and then decreased due to the effect of concentration quenching. Thus, 0.4 mol% of holmium concentration can be considered as optimum for the composition yHo_2_O_3_+(65-y)B_2_O_3_ + 15TeO_2_ + 10Pb_3_O_4_ + 10Na_2_O in the present study^49,51,99,100^.

Ho^3+^ ion-doped borotellurite glasses are superior compared to the silicate and phosphate glasses as they have low phonon energy, high refractive index, good rare earth ion solubility, and thermal stability. The Ho^3+^ ion-doped borotellurite glass systems have significantly higher refractive index (~ 1.9–2.35), compared to silicate (~ 1.65) and phosphate (~ 1.66) glass systems. The low phonon energy (~ 700–800 cm^−1^) of borotellurite glass host reduces the non-radiative loss and enhances the radiative transitions of Ho^3+^ ions. Their higher refractive index strengthens the local field, resulting in the increased emission intensity. Good network flexibility and better Ho^3+^ ion solubility allow for larger concentrations, leading to stronger and efficient luminescence, making them suitable for photonic and laser applications^52, 79,101-106^.

### CIE chromaticity diagram

**Fig. 20:**
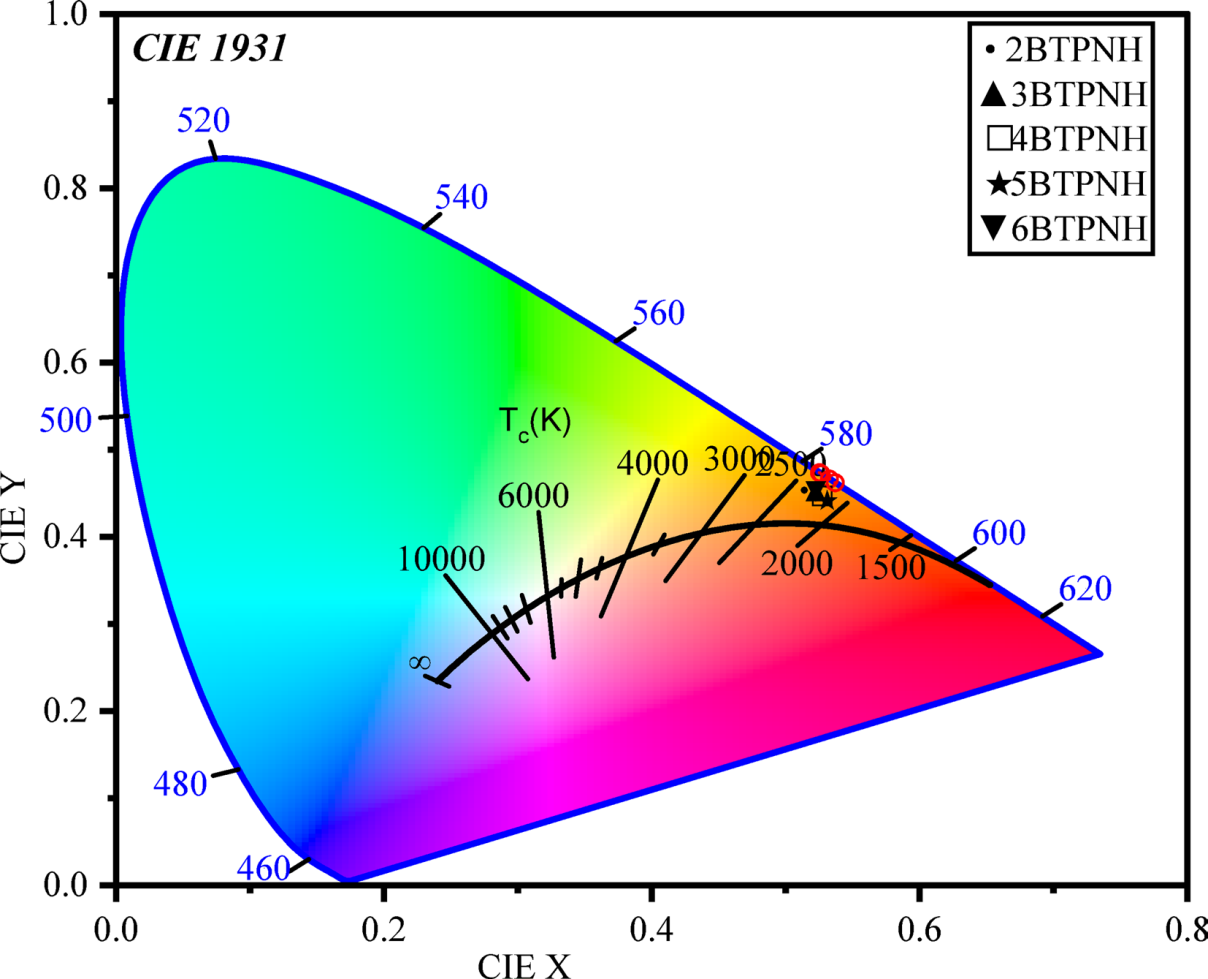
. CIE diagram of BTPNH glasses with different Ho_2_O_3_ (mol%) concentration.

In order to validate the emission color obtained for BTPNH glasses, a CIE (Commission Internationale de l’Eclairage) diagram was generated by utilizing the emission data and depicted in Fig. 20. The indicated points on the CIE diagram are in the Orange – Red region near the chromaticity edge. This verifies that the dominant emission color of these glasses is in the red spectral region, which is consistent with the intense 678 nm peak observed in the PL spectrum. The color correlated temperature (CCT), which highlights the temperature of Planckian black body radiators nearest to the operating point on the chromaticity diagram, ensured the quality of light. In Fig. 20, the black curve at the centre represents the black body (Planckian) locus, which represents the color of black body radiators at various temperatures. If the emission points are along this line, it indicates that the light appears more natural to the human eye. The emission from BTPNH glasses is slightly above this region, indicating a saturated red. The CCT is evaluated using coordinates by the McCamy formula^49^,

22 $$\:CCT=-449{a}^{3}+3525{a}^{2}-6823a+5520.3$$

Where, $\:a=x-0.332/y-0.186$, is the inverse line.

The estimated (x, y)- coordinates and CCT values for synthesized BTPNH glasses are listed in.

Table 6. The CCT values were found to be less than 4000 K, which indicates a warm CCT^49^. Based on the results and analysis, the BTPNH glasses are suitable for red phosphors, lasers, and optical amplifiers.

**Table 6:** CIE coordinates and CCT values.

Sample	(x, y) coordinates		CCT (K)
	x	y	
2BTPNH	0.525	0.473	2319.9
3BTPNH	0.526	0.472	2374.7
4BTPNH	0.532	0.466	2282.3
5BTPNH	0.536	0.461	2214.9
6BTPNH	0.526	0.472	2379.6

The lead oxide is advantageous in the structural and optical properties of glasses, it also causes environmental problems due to the toxicity of lead compounds. In the future, one can explore lead-free alternatives such as Bi_2_O_3_, ZnO, etc., in maintaining or enhancing the refractive index, thermal stability, and rare earth solubility in heavy metal oxide glasses. These alternatives will be evaluated for their capacity to maintain the current lead oxide-based glass system’s good optical performance for developing eco-friendly glass hosts for photonic and optoelectronic applications while ensuring environmental safety.

## Conclusion

The amorphous nature of the prepared BTPNH glasses was validated through the XRD technique. The vibrational modes as well as the relative proportion (N4) of BO_4_ in the examined glasses were investigated by ATR-FTIR spectroscopy. The structural modification and presence of the functional units BO_3_, BO_4_, TeO_3_, TeO_4,_ and Pb-O links in the glass network were confirmed through ATR-FTIR and Raman spectroscopy. SEM-EDX analysis showed a slightly inhomogeneous morphology and the elements B, O, Te, Na, Pb, and Ho in the synthesized glass. The density varies from 4.049 gcm^−3^ to 4.212 gcm^−3^. The direct band gaps were found between 3.115 eV and 3.201 eV. Electronic oxide polarizability and optical basicity are found in the range of 4.015 Å^3^ to 4.178 Å^3^ and 1.254 to 1.271, respectively. The metallization criterion was found to be between 0.35 and 0.45, which is suitable for optical devices. The 4f-4f electronic transition of Ho^3+^ ions, specifically ^5^F_5_ → ^5^I_8_, exhibits an intense red emission at 678 nm. The CCT values are $\:<$ 4000 K, which indicates a warm CCT. This signifies that BTPNH glasses are suitable for red luminescent devices. The structural modification induced by Ho^3+^ ions incorporation, tunable optical band gaps, and merits of physical and optical parameters, including density, optical band gap, electronic oxide polarizability, optical basicity, refractive index, metallization criterion, etc., suggest the obtained BTPNH glasses for lasers, red phosphors, and optical amplifiers.

## Data Availability

The data used and/or analysed during the current study available from the corresponding author on reasonable request.
